# Trends and disparities in non-communicable diseases in the Western Pacific region

**DOI:** 10.1016/j.lanwpc.2023.100938

**Published:** 2023-12-01

**Authors:** Wen Peng, Ling Zhang, Fuyuan Wen, Xiao Tang, Lingxia Zeng, Jiapeng Chen, Gauden Galea, Deliang Wen, Youfa Wang

**Affiliations:** a Nutrition and Health Promotion Center, Department of Public Health, Medical College, Qinghai University, People's Republic of China; b Department of Epidemiology and Health Statistics, School of Public Health, Capital Medical University, Beijing 100069, People's Republic of China; c The First Affiliated Hospital of Xi'an Jiaotong University Public Health Institute, Global Health Institute, School of Public Health, International Obesity and Metabolic Disease Research Center, Xi'an Jiaotong University, Xi'an 710061, People's Republic of China; d China Population and Development Research Center, No. 12 Dahuisi Road, Beijing, 100081, People's Republic of China; e WHO China Representative Office, No. 23 Dongzhimenwai Street, Beijing 100600, People's Republic of China; f Health Sciences Institute, China Medical University, No. 77 Puhe Road, Shenyang North New Area, Shenyang, Liaoning Province, People's Republic of China; g Qinghai Provincial Key Laboratory of Prevention and Control of Glucolipid Metabolic Diseases with Traditional Chinese Medicine, No. 16 Kunlun Road, Xining 810008, People's Republic of China; h Beijing Municipal Key Laboratory of Clinical Epidemiology, No. 10 Xitoutiao, Youanmenwai, Beijing 100069, People's Republic of China

**Keywords:** Non-communicable diseases, Trends, Disparity, Risk factors, The Western Pacific region

## Abstract

The WHO Western Pacific region bears disproportionate deaths from non-communicable diseases (NCDs), with increased overall NCD proportional mortality over the past two decades. The disease burden of mental health increased, resulting from rapid ageing, enhanced stress, and the COVID-19 pandemic, but it was largely neglected. The highly diverse cultures, religions, political systems, socioeconomic contexts, lifestyles, and environmental factors probably have led to massive disparities across countries in NCD mortality, risk factors, and NCD management. Geographically, East Asia had the lowest NCD mortality whilst Pacific islands had the highest. Economic booms, ageing, nutrition transition, social stress, prevalent tobacco use, and fast-increasing obesity and hyperglycaemia are important drivers of NCDs. Men tended to have more adverse behavioural and metabolic risk factors. Rural residents are catching up with their urban counterparts in metabolic risk factors and conditions. Sustainable strategies tailored to NCD patterns are needed to fight the NCD epidemic and related disparities.

## Introduction

Fighting non-communicable diseases (NCDs) is a public health priority worldwide and has particular relevance for the Western Pacific region. The United Nations has called on all countries to make a sincere effort to fight the growing NCD epidemic and included NCD prevention and control in the Sustainable Development Goal (SDG) 3.4.^1^ In concordance with global reported data, NCDs are the leading causes of death and disability in the region. Indeed, in this most populous area among the six WHO regions, the Western Pacific region data represent 1/4 of global NCD deaths. The four major NCDs–cardiovascular disease (CVD), cancers, chronic respiratory diseases, and diabetes–accounted for 12 million deaths in this region in 2019.^2^ The burden of mental health problems, as the fifth major NCD in the new 5 × 5 approach to NCDs favoured by the World Health Organization (WHO) in 2018,^3^ is relatively high and rising currently in the region.

The Western Pacific region distinguishes itself from other WHO regions by its large population, great diversity, and fast-growing economy. The region consists of 37 countries and areas (see Table 1), with nearly 1.9 billion total population. The geographical variance, multiple political systems and levels of socioeconomic development, complex ethnicities, languages, beliefs, cultures, lifestyles, and health statuses in this region probably have led to great disparities in NCD-related health outcomes across countries. Specifically, the gap in life expectancy was as high as 20.1 years in 2019 with the lowest 64.3 years in Tuvalu, and the highest 84.4 years in Japan.^4^ The mortality rates from NCDs also varied largely across countries in this region.^5^

**Table 1:** Profiles of 37 countries and areas in the Western Pacific region.

No.	Country/area	Population^a^	Year of Population^a^	GDP per capita ($)^b^	Year of GDP^b^	Income group^b^	Land area (km^2^)^b^	Ethnicity^c^	Major religion^c^
1	American Samoa (USA)	55,519	2010	12,844.90	2020	UMIC	2934	Pacific Islander (includes Samoan, Tangan), Asian 3.6%, mixed, other	Christian
2	Australia	23,717,421	2016	59,934.10	2021	HIC	7,692,020	English, Australian, Irish, Scottish, Chinese, other	Protestant, Roman Catholic
3	Brunei Darussalam	429,999	2021	31,722.40	2021	HIC	5270	Malay, Chinese, other	Muslim
4	Cambodia	15,552,211	2019	1591.00	2021	LMIC	5270	Khmer, Cham, Chinese, other	Buddhist
5	China	1,411,778,724	2020	12,556.30	2021	UMIC	5270	Han Chinese, ethnic minorities	Folk religion, Buddhist
6	Cook Islands	17,434	2016	–	–	–	–	Cook Island Maori (Polynesian), part Cook Island Maori, other	Protestant, Roman Catholic
7	Fiji	884,887	2017	5086.00	2021	UMIC	18,270	iTaukei, Indo-Fijian, Rotuman, other	Protestant, Hindu, other Christian, Roman Catholic, Muslim
8	French Polynesia (France)	281,674	2017	20,182.60	2020	HIC	3471	Polynesian, Chinese, local French, metropolitan French	Protestant, Roman Catholic
9	Guam (USA)	153,836	2020	34,624.30	2020	HIC	540	Chamorro, Filipino, White, Chuukese, Korean, other Pacific Islander	Christian, folk religions, Buddhist
10	Hong Kong SAR (China)	7,336,585	2016	49,660.60	2021	HIC	1050	Chinese, Filipino, Indonesian, and other	Buddhist or Taoist, Protestant, Roman Catholic, Muslim, Hindu, Sikh
11	Japan	126,226,568	2020	39,285.20	2021	HIC	364,500	Japanese, Chinese, Korean, and other	Shintoism, Buddhism
12	Kiribati	119,940	2020	1514.60	2020	LMIC	810	I-Kiribati, I-Kiribati/mixed, Tuvaluan, other	Roman Catholic, Kiribati Uniting Church
13	Lao People's Democratic Republic	6,492,228	2015	2551.30	2021	LMIC	230,800	Lao, Khmou, Hmong, Phouyhay, Tai, Makong, Katong, Lue, Akha, other	Buddhist
14	Macao SAR (China)	650,834	2016	45,421.60	2021	HIC	33	Chinese, Portuguese, mixed, other	Folk religion, Buddhist, Christian
15	Malaysia	28,334,135	2010	11,371.10	2021	UMIC	328,550	Bumiputera, Chinese, Indian, other	Muslim, Buddhist, Christian, Hindu
16	Marshall Islands	53,158	2011	4171.00	2021	UMIC	180	Marshallese, mixed Marshallese, other	Protestant, Roman Catholic
17	The Federated States of Micronesia	102,843	2010	3476.70	2021	LMIC	700	Chuukese/Mortlockese, Pohnpeian, Kosraean, Yapese, Yape outer islanders, Polynesian, Asian, other	Roman Catholic, Protestant
18	Mongolia	271,407	2020	4534.90	2021	LMIC	1,557,507	Khalkh, Kazak, Durvud, Bayad, Buriad, Zakhchin, Dariganga, other	Buddhist
19	Nauru	271,407	2011	12,252.30	2021	HIC	20	Nauruan, part Nauruan, I-Kiribati, other	Protestant, Roman Catholic
20	New Caledonia (France)	271,407	2019	272,620.00	2021	HIC	18,280	Kanak, European, Wallisian, Futunian, Tahitian, Indonesian, Ni-Vanuatu, Vietnamese, other	Christian
21	New Zealand	4,793,358	2018	48,801.70	2021	HIC	263,310	European, Maori, Chinese, Indian, Samoan, Tongan, Cook Island Maor, English, Filipino, New Zealander, other	Christian
22	Niue	1719	2017	–	–	–	–	Niuean, part-Niuean, non-Niuean	Ekalesia Niue, Church of Jesus Christ, Roman Catholic
23	Northern Mariana Islands	53,883	2010	20,659.60	2019	HIC	460	Asian, Native Hawaiian or other Pacific Islander, other	Christian
24	Palau	17,661	2015	14,243.90	2020	UMIC	460	Palauan, Filipino, Chinese, other	Roman Catholic, Protestant
25	Papua New Guinea	7,275,324	2011	2916.40	2021	LMIC	452,860	Melanesian, Papuan, Negrito, Micronesian, Polynesian	Protestant, Roman Catholic
26	Philippines	109,035,343	2020	3548.80	2021	LMIC	298,170	Tagalog, Bisaya, Cebuano, Llocano, Hiligaynon, Bikol, Waray, other local ethnicity, other foreign ethnicity	Roman Catholic
27	Pitcairn Island (UK)	45	2020	–	–	–	–	Descendants of the Bounty mutineers and their Tahitian wives	Seventh Day Adventist
28	Republic of Korea	51,829,136	2020	34,757.70	2021	HIC	97,600	Homogeneous	Protestant, Buddhist, Catholic
29	Samoa	195,979	2016	3939.10	2021	LMIC	2780	Samoan, Samoan/New Zealander, other	Protestant, Roman Catholic
30	Singapore	4,044,210	2020	72,794.00	2021	HIC	718	Chinese, Malay, Indian, other	Buddhist, Christian, Muslim, Taoist, Hindu
31	Solomon Islands	515,870	2009	2337.00	2021	LMIC	27,990	Melanesian, Polynesian, Micronesian, other	Protestant, Roman Catholic
32	Tokelau (New Zealand)	1285	2016	–	–	–	–	Tokelauan, part Tokelauan/Samoan, part Tokelauan/Tuvaluan, Tuvaluan, Kiribati, other	Congregational Christian Church, Roman Catholic
33	Tonga	100,651	2016	4624.80	2020	UMIC	720	Tongan, part-Tongan, other	Protestant, Church of Jesus Christ, Roman Catholic
34	Tuvalu	10,645	2017	5291.50	2021	UMIC	30	Tuvaluan, Tuvaluan/I-Kiribati, Tuvaluan/other, other	Protestant
35	Vanuatu	272,459	2016	3127.40	2021	LMIC	12,190	Melanesian, non-Melanesian	Protestant
36	Viet Nam	96,208,984	2019	3694.00	2021	LMIC	313,429	Kinh, Tay, Thai, Muong, Khmer, Mong, Nung, other	Catholic, Buddhist, Protestant, other
37	Wallis and Futuna (France)	11,562	2018	–	–	–	–	Polynesian	Roman Catholic

Income groups were per World Bank definitions in 2022; HIC: high-income economies. UMIC: upper-middle-income economies. LMIC: lower-middle-income economies.

a Data from the Demographic Statistics Database of the United Nations Statistics Division (https://unstats.un.org/unsd/demographic-social/products/dyb/#censusdatasets).

b Data from the World Bank (https://datahelpdesk.world_x0002_bank.org/knowledgebase/articles/906519-world-bank-countryand-lending-groups).

c Data from the Central Intelligence Agency of the United States (https://www.cia.gov/the-world-factbook/countries/).

To understand the complexity of the large NCD burden and related disparities in the Western Pacific region to inform strategies that could facilitate a healthier future, this study (see Panel 1 and Supplementary File 1) examined the trends and disparities in NCDs in this region and explored their related major risk factors at different levels.

Panel 1 **Search strategy and selection criteria**

We searched English (World Health Organization Global Health Data Exchange databases, PubMed, Web of Science, and Google Scholar), and Chinese databases (National Health Commission of the People's Republic of China, China National Knowledge Infrastructure, and Wanfang) from their inception to May 29, 2023, using the following search terms and their combinations: “NCD,” “non-communicable diseases,” “CVD,” “cardiovascular diseases,” “hypertension,” “obesity,” “overweight,” “BMI,” “diabetes,” “respiratory diseases,” “COPD,” “chronic obstructive pulmonary disease,” “cancer,” “tumor,” “neoplasms,” “nervous,” “neurological,” “mental health,” “psychiatric disorders,” “epidemiology,” “prevalence,” “awareness,” “treatment,” “control,” “Western Pacific region,” and names of all 37 countries and areas in the Western Pacific region. We also examined the reference lists of relevant literature and received advice on other data sources from experts on this research topic. Important publications and other data sources were included. Additionally, we systematically reviewed the published manuscripts about the prevalence and management of hypertension and diabetes in countries in the Western Pacific region from the online databases PubMed and Web of Science. We selected relevant studies by reviewing the titles, abstracts, and full texts. Flowchart of the included literature on hypertension prevalence and management is shown in Supplementary Fig. S1.

## Current status and trends of NCD mortality in the Western Pacific region

**Fig. 1:**
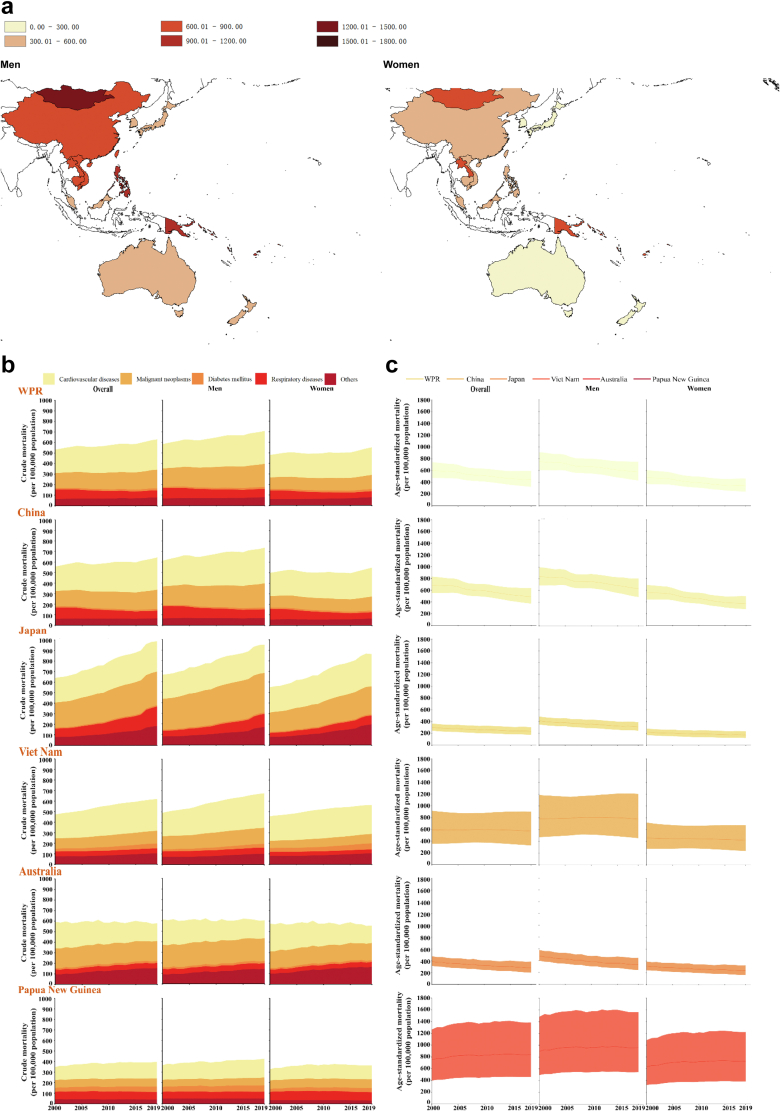
**Current status and trends of NCD mortality in Western Pacific region and its 37 respective countries/areas in the Western Pacific region.** a. Status in age-standardised mortality rates due to non-communicable diseases by sex in 21 countries and areas in the Western Pacific region in 2019. b. Compositional NCD crude mortality by sex in Western Pacific region and 5 select countries, 2000–2019. c. Overall age-standardised NCD mortality by sex in Western Pacific region and 5 selected countries, 2000–2019. Data on mortality rates were obtained from the World Health Organization Noncommunicable diseases: Mortality (https://www.who.int/data/gho/data/themes/topics/topic-details/GHO/ncd-mortality).

NCD mortality, as the endpoint of comprehensive NCD burden including NCD prevention and control, was selected as one of the most accurate measurements to assess successful and failed strategies. NCD mortality data is available for almost all countries and areas thus allowing for comparative analysis. Using WHO data,^6^ we described the status and trends of NCD mortality in the Western Pacific region, highlighted five selected countries (Australia, China, Japan, Papua New Guinea, and Viet Nam), compared and contrasted their conditions against global and other regions, and discussed their experiences and lessons (Fig. 1).

### Overall patterns of NCDs in the Western Pacific region

The four major NCDs in the Western Pacific region accounted for the vast majority and a disproportionately higher proportion of deaths as compared with global data (87.0% vs. 75.0%), and the proportion ranked the 2nd highest among all six WHO regions.^5^ The WHO data from the Global Health Observatory (Fig. 1A, data in Supplementary Table S1A) shows that men had overall higher age-standardised mortality from NCDs than women, which was in line with the global distribution by sex.^6^ The five lowest-ranking countries and areas in age-standardised mortality from NCDs in this region for both women and men were Australia, Japan, New Zealand, Singapore, and the Republic of Korea; while the highest-ranking for women were Federated States of Micronesia, Fiji, Kiribati, Solomon Islands, and Vanuatu, and for men were Federated States of Micronesia, Fiji, Kiribati, Mongolia, and Vanuatu.

Consistent with global trends and in other WHO regions, the proportional mortality from NCDs in the past decades in the Western Pacific region increased from 79.8% in the year 2000 to 87.4% in the year 2019 according to Global Burden of Disease (GBD) study results.^7^ Disaggregating the proportional mortality from CVD, cancers, and diabetes increased, and chronic respiratory disease decreased over time. Meanwhile, the crude NCD mortality continuously increased but age standardised NCD mortality decreased in this region (Fig. 1B and C, data in Supplementary Table S1B and C). This paradoxical phenomenon could be a result of the ongoing progress of the demographic transition towards an ageing population, and the epidemiological transition from communicable diseases to NCDs globally including in the Western Pacific region, particularly in low- and middle-income countries (LMICs) and countries in transition.^8^

### Conditions of NCDs in five selected countries

We selected five countries in the Western Pacific region to examine the status and trends in NCD mortality (Fig. 1B and C, data in Supplementary Table 2B and C). They are countries representative of the variation in stages of ageing, economic development, geographical/environmental characteristics, and health care systems and services. The selection includes two high-income countries (HICs) with a well-established health care system (Japan, with the highest average life expectancy and an ageing population, and Australia, with the majority of the population from European ancestral groups), one upper-middle-income country (UMIC) (China, the most populous country; rapidly ageing), and two LMICs (Viet Nam and Papua New Guinea). Alarmingly, Papua New Guinea, as a representative of the Pacific Islands, showed some reversal and a slightly increasing trend in age-standardised mortality. This unusual trend in this indicator suggested worsened NCD prevention and control in this country. The very high prevalence of obesity and diabetes and potentially prevalent unhealthy lifestyles, e.g., smoking in Pacific islands are potential risk factors.^9^^,^^10^ The interesting inverse association between the income groups and the width of the mortality confidence interval, particularly the large width in two LMICs, suggested the diverse degrees of data validity and reliability in NCD monitoring.

## Mental health in the Western Pacific region

Mental health is an important health issue in the Western Pacific region and has been added as the fifth major NCD in the new 5 × 5 approach to NCDs in the 3rd High-Level Meeting of the General Assembly on the Prevention and Control of NCDs.^3^ Over 215 million people in this region suffer from mental health conditions.^11^ The result of the 2019 GBD study showed that various mental health conditions, such as anxiety and depressive disorders, self-harm, schizophrenia, Alzheimer’s disease, and other forms of dementia significantly increased in the Western Pacific region from 1990 to 2019.^12^ In addition, emerging evidence has suggested that the COVID-19 pandemic had a massive negative impact on mental health and exacerbated this increasingly high public health issue.^13^

The universal increased disease burden due to mental disorders in this region was consistent with the global trends, which had an increased proportion of disability-adjusted life years (DALYs) from 3.1% in 1990 to 4.9% in 2019.^14^ The current status and trends in mental health were caused by the complex interactions of many multi-level factors. The increasing proportion of older people in this region as a result of a combination of longer life expectancies and decreasing fertility rates probably have led to the growing prevalence of dementia and other age-related mental health conditions.^15^ The rapid economic development, urbanisation, lifestyle transition, and related pressure in this region, may also lead to increased mental health conditions such as depression and anxiety.^16^ The impacts of the COVID-19 pandemic were substantial. High rates of psychological distress and signs of an increase in mental health disorders were observed, both as a result of the pandemic itself and strategies to cope with it, such as mitigation/containment and lockdowns/quarantines.^13^ Many countries, including China, have used digital health care in their mental health responses.^13^^,^^17^ However, this strategy also intensified the disparities in access to quality mental health care, particularly among older adults.^18^

Of note, from the GBD data, some HICs, e.g., Japan, Australia, and the Republic of Korea in this region have a higher disease burden from mental health than the LMICs, which was in line with the global distribution by income group.^14^^,^^19^ One potential reason may be due to internet overuse, which may increase the risk of online or cyber-bullying through various vehicles such as laptops and smartphones.^20^ The serious problem of suicide and school refusal among Japanese teenagers, and the derived social withdrawal among adults is a typical example.^21^ On the other hand, the potential drivers of disease burden from mental health in LMICs are heterogeneous in comparison with HICs. For instance, the high prevalence of sexual and interpersonal violence in LMICs may result in short- and long-term mental effects.^22^^,^^23^ Though the recognition of mental health problems in LMICs is growing, the gap still exists in monitoring the issue and studying the strategies, policies, and programs to prevent mental disorders by income groups.^24^ Barriers to mental health services and related stigma may hinder medical care-seeking behaviours, thus facilitating underestimating and increasing the disease burden. Potential barriers of mental health services in LMICs include the competing public health priority, the challenges in implementing population-based strategies in primary care settings, and insufficient numbers and limited types of mental health professionals and qualified community workers.^25^ Stigma of mental disorders is common in almost every country, and impacts people living with this condition, their families and health professionals. Certain prerequisites are necessary for addressing and overcoming stigma adequately, including comprehensive and integrated mental health policies and legislation, sustainable and culturally adapted intervention programs, capacity building of mental health and other workforce professionals, and the integration of mental health services into primary care settings. However, these conditions are largely inadequate in LMICs.^26^ Cultural factors (e.g., collectivism, Confucianism, concern over saving face, religion, and supernatural beliefs) are potential socio-cultural determinants that facilitate the hesitation for mental health acknowledgement and medical care-seeking behaviours.^27^

Within countries, disadvantaged populations are commonly disproportionately affected by poor mental health conditions resulting from higher exposure to stress due to poverty, disparities in access to mental health services, and low health literacy.^14^^,^^19^ A substantial lack of resources for sufficient access to medical care may prevent disadvantaged people from care-seeking behaviours.^24^

## Disparities in NCD mortality by selected macro-level factors in the Western Pacific region

**Fig. 2:**
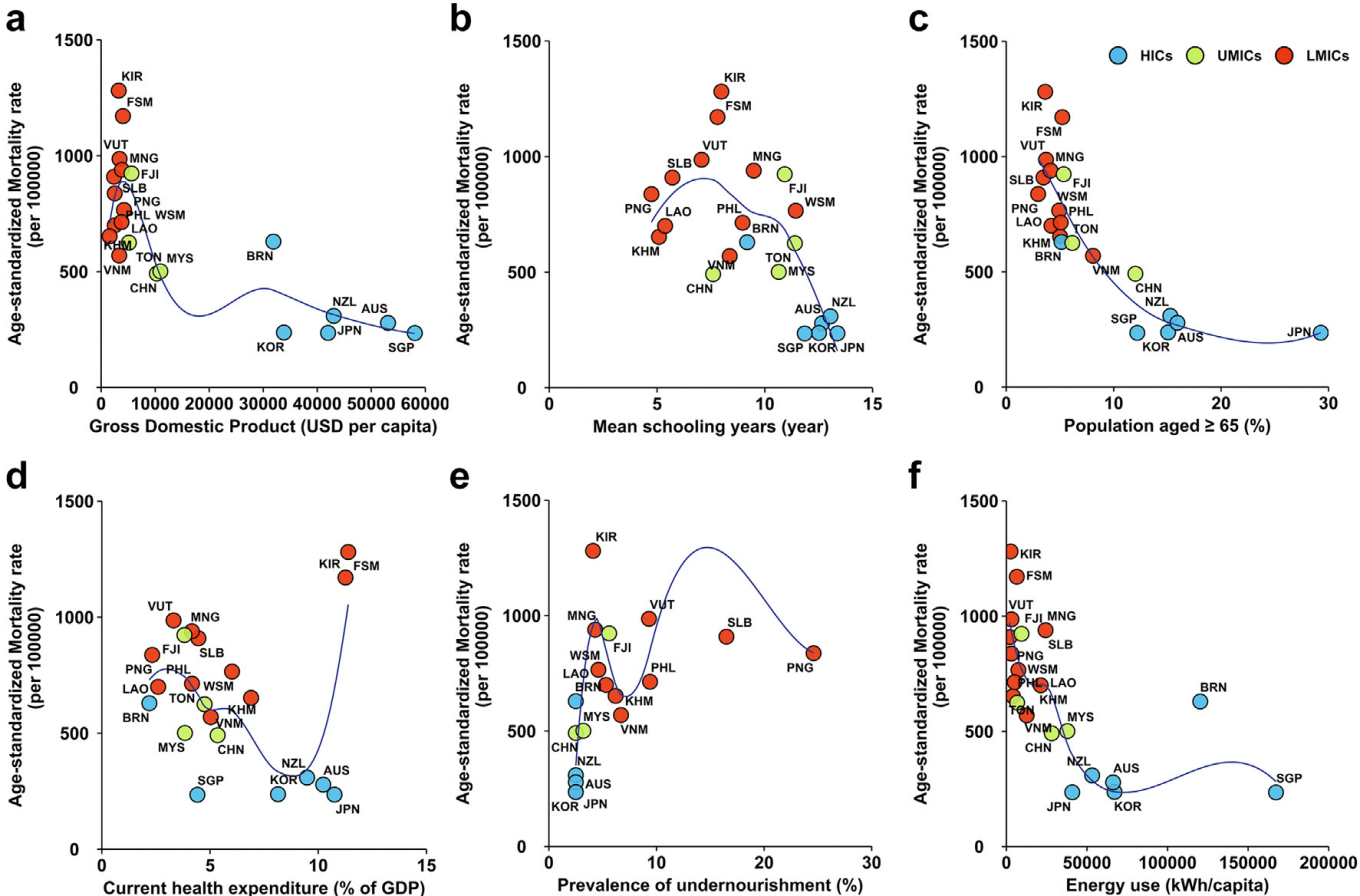
**Association between age-standardised mortality rates due to non-communicable diseases and potential risk factors in 21 countries and areas in the Western Pacific region in 2019.** a. gross domestic product; b. Mean year of schooling; c. Percentage of the population aged ≥65; d. Current health expenditure; e. Prevalence of undernourishment; f. Energy use per capita. Data on age-standardised mortality rates were obtained from the World Health Organization Noncommunicable diseases: Mortality (https://www.who.int/data/gho/data/themes/topics/topic-details/GHO/ncd-mortality). Data on the risk factors were obtained from the World Bank Open Data (https://data.worldbank.org). Each point represents one country or area, and countries and areas are colored by region. USD: United States dollars; HICs: high-income countries; UMICs: upper-middle-income countries; LMICs: lower-middle-income countries.

Large disparities existed in the distribution of both the NCD mortality and potential socioeconomic and other related risk factors (Fig. 2). The highest age-standardised mortality from NCDs was 1281.0 per 100 thousand population in Kiribati, which was 5.4 times higher than the lowest country (234.8 per 100 thousand population in Singapore). Socioeconomic (gross domestic product (GDP) per capita and education), demographic (percentage of aged population), health expenditure (current health expenditure as a percentage of GDP), and some other related factors listed in Fig. 2 using World Bank Open Data,^28^ all varied widely. Some risk factors are aggregated by different types of features such as income group, geographic, or cultural factors. Both the trends of the fitting curve and the aggregation pattern of the potential risk factors by income groups are of interest. HICs had more schooling years, a higher percentage of GDP as health expenditure, a higher percentage of the aged population, higher energy use per capita, and a lower prevalence of undernourishment compared with UMIC and LMICs. Consequently, HICs also had lower age-standardised NCD mortality.

An overall inverse association was observed between age-standardised mortality from NCDs and indicators or proxies of socioeconomic development, including GDP per capita, mean schooling years, and energy use per capita (Fig. 2A, C, and F). This is consistent with the conditions globally and in other regions,^5^ indicating such social determinants of health may have the strongest impacts on health outcomes. The Pacific islands had the highest age-standardised mortality from NCDs. Further, most Pacific islands were above the fitting curve in Fig. 2D, indicating lower health system efficiency, with higher health expenditure but only limited performance of NCD prevention and control in these countries and areas. The very high obesity rates in the Pacific islands, which is an important intermediate and monitoring indicator for NCD prevention and control, may be an important reason as discussed before.^9^^,^^29^^,^^30^ By contrast, some countries below the fitting curve in Fig. 2D had higher health system efficiency by using relatively limited health expenditure but achieved better performance, e.g., Singapore, the Republic of Korea, Japan, China, and Malaysia.

A broader overview of the global conditions shows the general gradient in health and socioeconomic status across and within countries: lower socioeconomic position, worse health.^31^ Despite overall limited studies, such evidence tended to accumulate in HICs, such as European countries,^32^ and was scarce in UMICs and LMICs. This was probably associated with the competing resources for research topics in UMICs and LMICs, where studies on health disparities were not prioritised. Some outlier examples with inferior economic development but outstanding performance in health outcomes provided some promising insights for overcoming the health divide related to the economic status through improving health system performance. Cuba had a life expectancy at birth of 78 years in 2020, which was comparable to many HICs.^28^ The divergence in health and economic indicators for Cuba was even more pronounced in the 1990s. Cuba's health system represents an example of a national integrated approach resulting in improved health for the mass population. The development of family medicine and tax-financed universal health systems in Cuba enabled equitable access to quality care.^33^^,^^34^ Such successful health system performance provided invaluable experiences for other countries, particularly for those with limited resources.

Minority population is also vulnerable and demonstrates different patterns in NCDs and their related risk factors by income groups. In Australia, the HIC, the prevalence of obesity and diabetes were higher in indigenous people than that in general population (obesity/overweight 74.9% vs. 64.3%; diabetes 12.6% vs. 4.3%).^35^ In China, the UMIC, a study in 2014–2015 included more than 726 thousand participants showed higher prevalence of overweight/obesity in Uyghur (65.5%), Manchu (38.0%), and Molgol people (34.3%) and lower obesity prevalence in Muslim (27.1%) than Han people (30.6%).^36^ Other national surveys in China suggested major minority population had lower prevalence of hypertension and diabetes but also lower awareness, treatment and control rates of both conditions compared to the Han population.^37^, ^38^, ^39^, ^40^

The generally low prevalence of some NCD metabolic risk factors, e.g., obesity and hypertension, contributed to the low mortality from NCDs in some countries. Japan and the Republic of Korea are the two countries with the lowest obesity rates in HICs worldwide (4.4% and 4.9%, respectively).^41^ The actions taken to reduce other risk factors, such as smoking cessation and the promotion of a healthy diet in some Eastern Asian countries, may also play important roles in reducing NCD burden.^42^^,^^43^ Thus, policy and interventions targeting the five major NCD risk factors (tobacco use, unhealthy use of alcohol, poor diet, inadequate physical activity, and environmental pollution) and controlling the metabolic risk factors (overweight and obesity, increased blood pressure, raised blood glucose, and dyslipidemia) should be prioritised. From the health economics perspective, promoting a healthy lifestyle is the most cost-effective investment in population-level health.^44^

Ageing is an important risk factor for NCDs. The seemingly contradictive inverse association between NCD age-standardised mortality and the percentage of the population aged ≥65 years (Fig. 2C) was explained by that countries in aged societies were more likely to be socioeconomically advanced, which is a strong predictor for low age-standardised mortality from NCDs.

## Indicators for NCD management in the Western Pacific region and selected countries

The prevalence, awareness, treatment, and control rates of hypertension and diabetes are important indicators for NCD management and are included in the WHO NCD monitoring framework. The suboptimal conditions and disparities in NCD prevalence and management, indicated by the low rates of and large gaps in these rates of hypertension and diabetes, need to be addressed in the future, including national policies and programs.

**Fig. 3:**
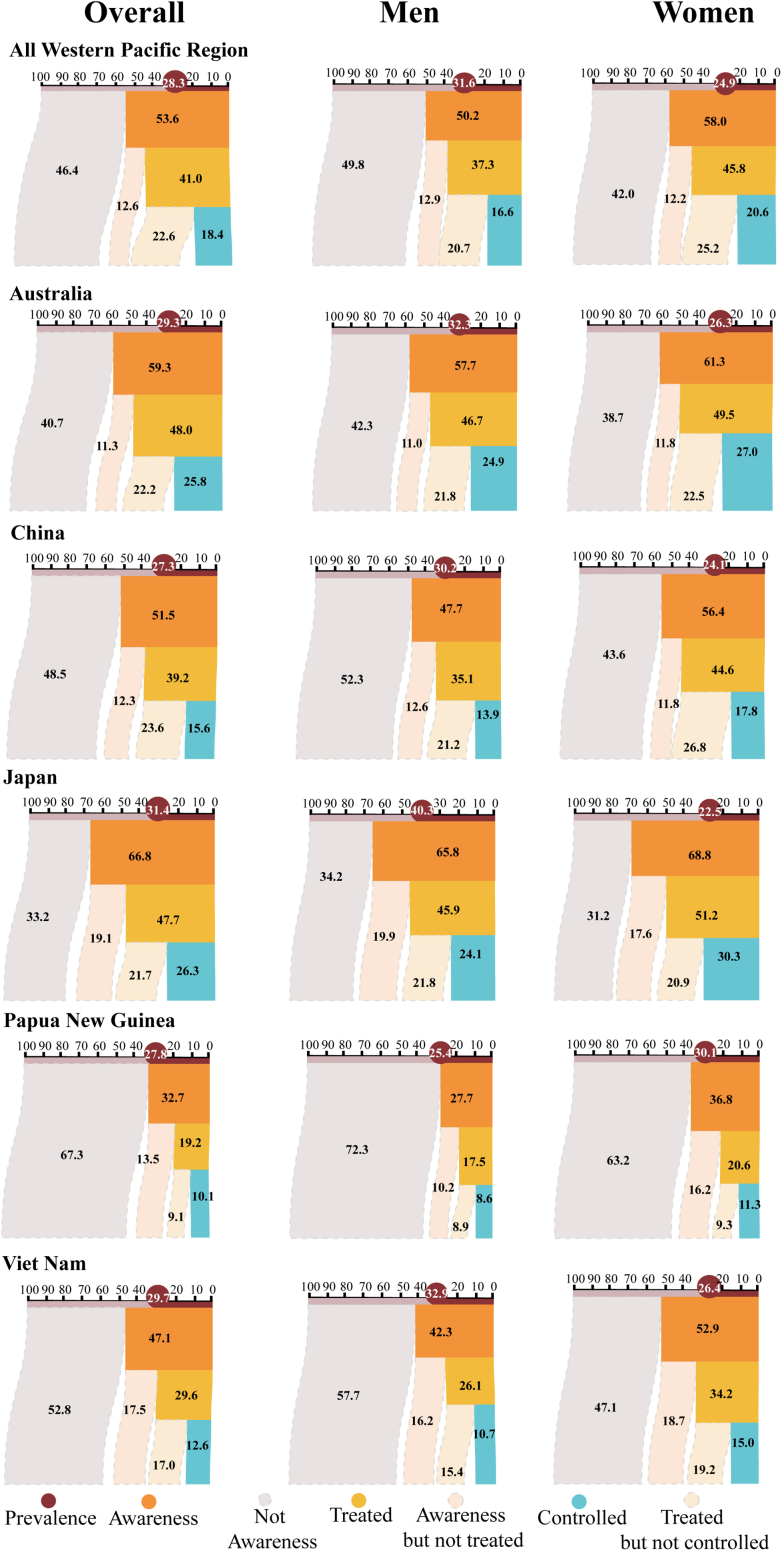
**Prevalence, awareness, treatment, and control rates of hypertension in overall Western Pacific region and selected countries in the Western Pacific region.** Data on the prevalence, awareness, treatment, and control rates of hypertension were obtained from the World Health Organization Noncommunicable diseases: Risk factors (https://www.whoi.nt/data/gho/data/themes/topics/noncommunicable-diseases-risk-factors).

To address this concern, we systematically reviewed studies on the prevalence and management rates of hypertension and diabetes based on nationally representative surveys in the Western Pacific region and respective 37 countries and areas. The efforts identified limited studies on hypertension (Supplementary Table S2) and a few on diabetes. Thus, we presented the key results for the overall Western Pacific region and five selected countries (see Fig. 1). We selected data sources for comparability across countries from the WHO NCD database,^6^ detailed in Fig. 3 and Table 2.

**Table 2:** Disease burden and management (%) of diabetes in representative countries in the West Pacific region.^c^

Countries	Year	Diagnostic criteria	Age (year)	Outcomes (%)			
				Prevalence	Awareness	Treatment	Control
Japan^45^	2021	ADA 2018	20–79	All 6.6	All 54.5^a^	/	/
	2013–2017	/	40–74	/	/	All 79.9	All 55.3
Australia^46^^,^^47^	2021	ADA 2018	20–79	All 6.4	All 75.0^a^	/	/
	2020–2021	/	≥15	/	/	All 76.1	/
	2015	/	≥18	/	/	/	All 28.0^b^
Viet Nam	2020	ADA 2018	20–79	All 6.1	All 48.5^a^	/	/
China^48^	2021	ADA 2018	20–79	All 10.6	All 48.3^a^	/	/
	2018	ADA 2018	≥18	/	/	All 32.9	All 50.1
Papua New Guinea	2021	ADA 2018	20–79	All 16.7	All 48.5^a^	/	/
Western Pacific Region	2021	ADA 2018	20–79	All 9.9	All 47.2^a^	/	/

a Awareness rate was calculated by 1-proportion of patients with undiagnosed diabetes.

b Control rate calculation included only type 2 diabetes.

c Data from the International Diabetes Federation (for prevalence and awareness rates; https://diabetesatlas.org/data/en/indicators/2/), and multiple authoritative sources (for treatment and control rates).^45^, ^46^, ^47^, ^48^

In the Western Pacific region, the status and disparities in prevalence and management rates of hypertension and diabetes suggested a major challenge for NCD prevention and control. The prevalence of hypertension in the overall Western Pacific region and the five selected countries did not have large variability. All were high, ranging from 27.3% in China to 31.4% in Japan. However, the rates of awareness, treatment, and control of hypertension were generally low and varied largely across countries. Further, the data showed an inverse association between income and the management rates of hypertension. Among these five countries, the control rates were as low as around 10% in Papua New Guinea and Viet Nam, and only about 1/4 as that in Australia and Japan. Generally, men had a higher prevalence of hypertension than women, while women had higher awareness, treatment, and control rates than men (Fig. 3).

The limited studies available on diabetes revealed generally low levels of and large disparities in prevalence and management rates across countries. Papua New Guinea had the highest prevalence of diabetes of 16.7%, and Australia had the highest awareness rate of 75.0% (Table 2). No nationally representative or comparable data was found for the difference by sex.

Evidence on time trends in NCD management also showed continuously prevalent hypertension and diabetes, but stagnated rates and uncontrolled situations in both conditions in the past two decades in the Western Pacific region.^49^^,^^50^ With China as an example, the prevalence of hypertension increased from 18.8% in 2002 to 25.2% in 2012 and 27.5% in 2018, and the prevalence of diabetes increased from 2.6% in 2002 to 10.4% in 2012 and 11.9% in 2018. In this same period, the awareness, treatment, and control rates of both conditions had a modest improvement, but the rates remained low. Specifically, the control rate for hypertension in China was as low as less than 20%, and the rate for diabetes was about 1/3 in 2018.^51^ Of importance, rural residents were more adversely affected and had higher increase rates in prevalence of both conditions compared with urban residents,^48^^,^^50^^,^^51^ suggesting the prominent phenomenon of disparity in NCD management by socioeconomic status within countries. Similar intra-country disparities can also be observed in other countries by sex, income, education, occupation, ethnicity, religion, etc.^52^ The easily neglected social determinants of health need to be explored to address health inequities for vulnerable populations.^53^

## Determinants of NCD burdens in the Western Pacific region

**Fig. 4:**
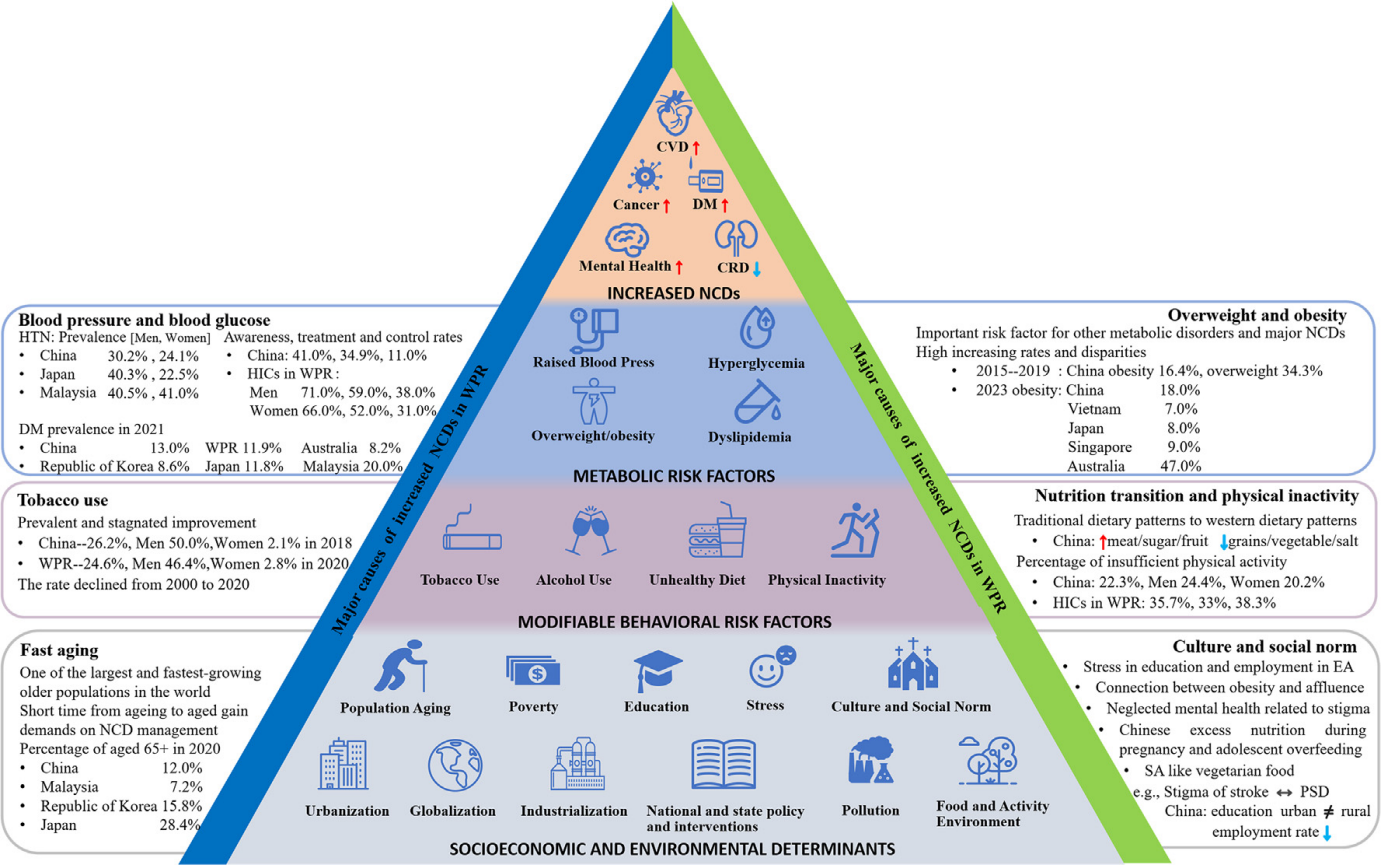
**Major causes and drivers of increased non-communicable diseases in the Western Pacific region.**^29^^,^^54^, ^55^, ^56^, ^57^, ^58^, ^59^, ^60^, ^61^, ^62^, ^63^, ^64^, ^65^ Describe the major causes and drivers that contribute to non-communicable diseases. CRD, Chronic Renal Disease; CVD, Cardiovascular Disease; DM, Diabetes Mellitus; HICs, high-income countries; HTN, hypertension; M, men; NCD, non-communicable diseases; PSD, post-stroke depression; W, women; WRP, Western Pacific region.

The rapid increase in the NCD disease burden in the Western Pacific region is a result of complex interactions of many determinants in multiple domains. Fig. 4 shows the framework of three domains of potential determinants of increased NCDs and some unique examples in the Western Pacific region. We developed this framework based on the Regional Action Framework for Noncommunicable Disease Prevention and Control in the Western Pacific and findings from other related studies.^66^, ^67^, ^68^ Three consecutive bottom-up layers of determinants are listed in a pyramid: socioeconomic and environmental determinants, modifiable behavioural risk factors, and metabolic risk factors.

### Mutual and reciprocal impacts between determinants and increased NCDs

The complex interaction and mutual-reinforcing effects of these determinants have an important collective impact on increased NCDs.^66^^,^^67^ Reciprocally, the increased NCD burden can exacerbate socioeconomic status by increased healthcare-related costs and decreased labor capability of patients with NCDs. In particular, disadvantaged populations with inadequate resilience and inferior capability of coping with such stress, will suffer more and may sink into a reciprocally vicious cycle unless necessary aid and other timely intervention programs are provided.^69^, ^70^, ^71^ A national survey of 29,712 poor households in rural China in 2017 showed that 51.6% of households attributed their poverty to diseases, among which most were NCDs.^72^ During the COVID-19 pandemic, patients with NCDs were particularly vulnerable to increased and excessive morbidity and mortality.^73^ Countries and areas with an inadequate capacity to cope with the COVID-19 pandemic suffered more, thus further increasing NCD-related poor health outcomes.

### Socioeconomic and environmental determinants

Some unique socioeconomic and environmental factors in the macro-level determinants of NCDs existed in the Western Pacific region. Fast ageing is one of the most prominent ones. Unlike most European countries, which took more than 60 years to finish the transition from ageing to an aged society, the time for most Asian countries in the Western Pacific region is predicted to be about only 15–25 years, regardless of the income group. Examples include both HICs and LMICs such as Japan (24 years), the Republic of Korea (18 years), and Singapore (17 years), China (23 years), Viet Nam (18 years), Malaysia (24 years), and Mongolia (25 years).^74^ In the Western Pacific region, China has the largest number of older adults aged 65 and older globally, totaling 200.6 million and accounting for 14.2% of the total population in 2021^75^; Japan has the largest percentage of the aged population of 29.3% in 2021 (Monaco excluded) and this proportion will continue to increase.^28^

In addition, some cultural and social norms in this region also contributed to the increased NCD burden but these factors have been understudied. Specifically, the common traditional perception of the close connection between obesity and affluence in some Asian countries, which has been examined in South Asian countries, such as India, Pakistan, and Bangladesh,^76^ were rarely studied in the Western Pacific region. The topic of stigma and mental health has only very limited epidemiological data with large uncertainty intervals, without further exploration for potential key effective interventions.^27^ The culture-related perfectionism mindset and the consequent intensive competition-related stress in education and job markets in East Asian countries, e.g., China, Japan, and the Republic of Korea, are perhaps interesting cultural determinants and were considered important drivers for the decreased fertility rates in these countries,^77^ but are largely understudied. The tendency of study aggregation on the biological pathway rather than the macro-level determinants of NCDs, which may have important impacts on policy and intervention programs, highlights the research gap in NCDs in this region.

Furthermore, the increased NCD burden may also be attributed to environmental determinants such as air pollution and climate change.^78^ To be specific, the adverse effect of air pollution exposure on NCDs was mainly reported among individuals in East Asian countries, such as China, Korea, and Japan.^79^ Pacific islands are areas that suffered most from the elevated sea level resulted from climate change. Some islanders are forced to migrate from their homeland to other places. Related anxiety and other mental disorders should be investigated further.

The socioeconomic and environmental determinants had universal impacts on the overall population, but some detrimental influences affected the disadvantaged more adversely. Some contextual risk factors, such as the unhealthy food and activity environment and the negative impacts of the global food industry affected the poor and disadvantaged most.^69^^,^^71^ The Western Pacific region presents significant disparities in levels of socioeconomic status across and within countries. The GDP per capita ranged from 72794.0 USD in Singapore to 1606.5 USD in Kiribati in the year 2021. The Gini Index, an indicator for measuring the equity of income distribution in a country, reached 42.3 in the Philippines in 2018, higher than the value of 41.4 in the United States in the same year.^28^^,^^53^^,^^80^ Such social determinants of health should be addressed and more efforts should be taken to tackle these potential health equity issues in this critical time window of a transitional period in many countries and areas in this region.

### Behavioral and metabolic risk factors

The prevalence of behavioral and metabolic risk factors is an important pointer to the NCD burden. Among the modifiable behavioral risk factors, the nutrition transition from traditional to western diets, driven by the food system transformation after urbanisation and globalisation, is happening in many countries in the Western Pacific region in the past decades, regardless of income groups, such as in the Republic of Korea, China, and Pacific islands.^54^^,^^81^^,^^82^ Historically, most countries in the region have suffered from wars, conflicts, and unrest over the past decades: the Second World War, Viet Nam War, and Korean War. From the life course nutrition perspective, food insecurity and famine related to these situations may have bred an increased risk of obesity and related metabolic disorders and NCDs in this region, particularly in the context of nutrition transition.^83^ These unique historical contexts were suggested to have contributed to the fast-increasing prevalence of elevated blood glucose (diabetes prevalence 1–2% in the early 1980s to around 12.5% in 2021 in adults) and the current uniformly high prevalence of raised blood pressure (all over 25% and country-specific prevalence from 25% to 50%) and dyslipidaemia (all over 25%) in this region.^84^^,^^85^

Commercial determinants are potential underlying causes for the behavioural risk factors including the nutrition transition and other factors. Resulted from colonialism and commercial fishing, the Pacific islands had limited local access to fishing and replaced local food stuffs with processed food and animal products.^86^ The global and local corporations’ commercial promotion for tobacco, alcohol, sugar-sweetened beverages, and fast food, together with the reluctant willingness of governments to intervene in these areas through extensive public health campaigns or legally, e.g., by financial or tax measures, have also contributed to the prevalent behavioral risk factors.^87^ Such examples include the prevalent and stagnated smoking rates in China and Viet Nam, particular in men (50.0% in Chinese men in 2018, and 48.0% in Vietnamese men in 2015)^51^^,^^88^ and the increasing consumption of sugar-sweetened beverages in many LMICs and the over-recommended-daily-limit consumption for free sugar in many HICs.^89^

Disparities in behavioural and metabolic risk factors also existed in the Western Pacific region. Men tended to have more adverse behavioural factors (e.g., tobacco use and alcohol consumption) and metabolic risk factors (e.g., raised blood glucose and increased blood pressure). Low socioeconomic groups usually had a significantly higher prevalence of unhealthy behaviours, e.g., unhealthy diet, tobacco use, than high socioeconomic groups in both HICs and LMICs.^90^ Behavioural modification is also very challenging for the vulnerable mostly due to social determinants of health, such as difficulties in physical and economic access to healthy food and to safe spaces for exercise, such as the case in Papua New Guinea.^91^ Metabolic risk factors were also more prevalent among urban residents in LMICs and transitional countries, but the differences by residence were closing.^55^^,^^80^^,^^85^ Data in China showed that the prevalence rates of current smokers (50.0% vs. 2.1%), excessive drinking (17.6% vs. 3.7%), hypertension (30.8% vs. 24.2%), and diabetes (12.9% vs. 10.9%) in men were all higher than in women; further, the rates for obesity (17.5% vs. 15.3%), diabetes (12.6% vs. 11.1%) and dyslipidaemia (36.5% vs. 34.6%) were all higher in urban than in rural residents, but the rates for hypertension were reversed (25.7% vs. 29.4%) in 2018.^51^ Interestingly, the top five countries with the highest prevalence of obesity and those with the highest prevalence of diabetes were all Pacific islands.^9^^,^^85^ The geographic aggregation of metabolic risk factors is interesting and the underline reasons and tailored interventions need to be explored further.

## Highlights of intervention efforts in China

The Western Pacific and European regions are the only two WHO regions predicted to achieve SDG 3.4 targets on NCDs by 2030.^92^ With the largest population and the second largest economy globally, China has made good progress overall NCD prevention and control and heavily influenced the overall progress in premature mortality due to NCDs in this region. Some successful practices in China can provide useful insights for other countries.

During recent years, the Chinese government has promoted comprehensive population-based strategies to improve public health, launched several landmark national programs and enacted several laws. In 2016, China announced the “Healthy China 2030” national initiative; in 2019, it launched the “Healthy China Action Plan (2019–2030)”.^93^ Campaigns to promote health, such as National Nutritional Week held annually since 2015^94^ and China’s ‘Healthy Lifestyles for All’ held since 2007,^95^ have made some major contributions to health lifestyle.

Obesity is a good indicator and intervention target of lifestyle risk factors of many NCDs. In October 2020, China released the “Action Plan to Prevent and Control Obesity among Children and Adolescents”. Our team published the comprehensive “Blue Paper on Obesity Prevention and Control in China” in 2019 and in a 2021 paper we outlined policy recommendations for China to fight the growing obesity epidemic.^12^^,^^96^ We have carried out a series of academic and community-based intervention efforts to fight against obesity as part of the March 4th World Obesity Day global efforts since 2022.

Moreover, community-based hypertension and diabetes management programs in China helped in improving NCD management. Healthcare system reforms, including universal healthcare coverage, enhanced financial security, and optimised health care resource allocation, have also improved the efficiency and productivity of the health care system, thus helping to improve NCD prevention and control.^97^

On the other hand, the stagnated improvement or even worsened conditions in some behavioural risk factors for NCDs and the deterioration in metabolic risk factors in China also called on for more efforts. In China, 50% of men smoke and almost 20% of men had excessive alcohol drinking, with smoking declining very slowly and alcohol consumption slowly increasing in the past two decades. The prevalence of overweight and obesity has been rising, and hypertension and diabetes largely were uncontrolled.^51^ To promote healthy lifestyles and appropriate management of NCDs are urgently needed in China, as well as in many other countries and areas in the Western Pacific region.

## Recommendations to cope with NCDs in the Western Pacific region

We recommend several strategies to cope with the increasing NCD burden and related disparities in this region. First, “active health” and health-centred concepts should be promoted for long-term health. This includes more vigorous effects on promoting health lifestyle, and better programs on screening and self-management among high-risk populations and NCD patients. Second, countries should implement programs on prevention, early detection, management, and reducing social stigma about mental health. These include to enhance mental health services (its accessibility and quality), professional training and quality health facilities. Third, some cultural and social norms deserve increased attention. We need comprehensive research to understand health perceptions. Perfectionism-related stress should be explored and mitigated through wellbeing initiatives. A holistic research approach to NCDs considering macro-level determinants is critical. Policies addressing harmful norms and promoting health equity should be implemented. Interdisciplinary collaboration, community engagement, and local health system capacity strengthening are key to a holistic, culturally sensitive, and evidence-based approach to NCDs. Finally, more sustainable and effective efforts are needed to support vulnerable populations, such as those living in LMICs and those with low socioeconomic status, to decrease health disparities.

All above strategies require collaborative efforts from governments, healthcare providers, communities, and individuals and should be customised according to the specific circumstances of each country.

## Conclusions

The NCD burden increase over the past decades and disparity in NCDs is common across and within countries in the Western Pacific region. Behavioural risk factors stagnated, metabolic risk factors continuously increased, and macro-level determinants were understudied. The prevalence of major NCDs is high while the rates of awareness, treatment and control are low in some countries in the region where data are available. Varying and sustainable coping strategies depending on changes in NCD patterns, and risk factors are needed for countries to combat NCDs in this region.

## Contributors

YW, WP, and LZ initiated the concepts; LZ, FW, and XT reviewed the data and drafted figures and tables; YW, WP, LZ, FW, and XT conducted literature review; and YW, WP, and LZ drafted the manuscript. All authors revised the manuscript critically, and approved the submission of the manuscript in its current form.

## Editor note

The Lancet Group takes a neutral position with respect to territorial claims in published maps and institutional affiliations.

## Declaration of interests

The authors declared no conflict of interest.

## Appendix ASupplementary data

Supplementary Material

Translated Abstract
