# Supplementary material for: Trends and disparities in non-communicable diseases in the Western Pacific region
Source: Lancet Reg Health West Pac. 2023 Dec 1;43:100938. doi: 10.1016/j.lanwpc.2023.100938 (PMC10920054; doi:10.1016/j.lanwpc.2023.100938)
Supplement: Supplementary Material [file mmc1.docx]

**Trends and disparities in** **non-communicable diseases in the Western Pacific region**

**Supplementary file 1: Data Source and Study Methodology**

This study made use of data from various sources such as the data on mortality rates of overall non-communicable diseases (NCDs), cardiovascular disease (CVD), cancers, chronic respiratory disease (CRD), and diabetes from 2000 to 2019 obtained from the World Health Organization (WHO) Noncommunicable diseases (<https://www.who.int/data/gho/data/themes/noncommunicable-diseases>) to observe the current status and trends of the disease burden. Data on the prevalence, awareness, treatment, and control rates of hypertension were obtained from the WHO NCD database: Risk factors (https:/www.who int/data/gho/data/themes/topics/noncommunicable-diseases-risk-factors) to show the status and disparities in prevalence and management rates of hypertension. Data on the potential risk factors were obtained from the World Bank Open Data (<https://data.worldbank.org>) to assess the association between age-standardized mortality rates due to NCDs and these risk factors.

The completed data for the Western Pacific region (WPR) overall and a total of 21 countries (Australia, Brunei Darussalam, Cambodia, China, Fiji, Japan, Kiribati, Lao People's Democratic Republic, Malaysia, Micronesia, Mongolia, New Zealand, Papua New Guinea, Philippines, Republic of Korea, Samoa, Singapore, Solomon Islands, Tonga, Vanuatu, Viet Nam) were available and analyzed.

All analyses and visualization were performed using R 4.2.2. The distribution of age-standardized NCD mortality rates in different countries and areas was shown by map graphs. Line graphs were performed to show the trends of NCD and its specific composition mortality from 2000 to 2019. Scatter graphs with a smooth line based on locally estimated scatterplot smoothing (LOESS) were applied to present the association between age-standardized mortality rates for NCD and potential risk factors. Sankey graphs were used to show the prevalence, awareness, treatment, and control rates of hypertension in overall WPR and selected countries (Australia, China, Japan, Viet Nam, and Papua New Guinea).

**Supplementary Tables**

**Table S1A. Current status and trends of NCD mortality in WPR and countries/areas in the Western Pacific region in 2019.**

| **Country/area** | **Both** | **Men** | **Women** |
| --- | --- | --- | --- |
| Western Pacific region | 443.8 (323.9, 593.2) | 574.1 (430.0, 749.2) | 569.4 (326.9, 900.9) |
| Australia | 278.5 (197.5, 385.1) | 328.4 (239.4, 447.9) | 337.0 (239.0, 462.4) |
| Brunei Darussalam | 629.5 (419.9, 912.7) | 645.4 (434.5, 925.3) | 233.5 (160.2, 327.7) |
| Cambodia | 652.4 (379.4, 1045.0) | 791.8 (465.6, 1254.0) | 612.5 (404.9, 897.9) |
| China | 491.5 (370.8, 638.9) | 635.6 (490.2, 806.9) | 555.4 (320.2, 897.8) |
| Fiji | 923.4 (600.9, 1351.0) | 1085.0 (716.7, 1571.0) | 372.7 (274.5, 497.2) |
| Japan | 235.4 (177.3, 304.2) | 310.7 (245.6, 389.0) | 788.9 (504.0, 1168.0) |
| Kiribati | 1281.0 (737.3, 2062.0) | 1531.0 (890.7, 2433.0) | 174.3 (122.6, 235.1) |
| Lao People's Democratic Republic | 699.9 (411.6, 1119.0) | 805.4 (472.6, 1283.0) | 1095.0 (626.6, 1773.0) |
| Malaysia | 501.1 (309.9, 773.1) | 558.1 (352.3, 847.7) | 610.1 (360.5, 978.4) |
| The Federated States of Micronesia | 1171.0 (594.8, 1938.0) | 1327.0 (670.5, 2179.0) | 444.2 (267.7, 698.2) |
| Mongolia | 939.2 (581.7, 1440.0) | 1231.0 (774.0, 1858.0) | 1027.0 (529.4, 1708.0) |
| New Zealand | 308.9 (225.6, 416.0) | 354.3 (265.2, 470.6) | 721.3 (442.4, 1120.0) |
| Papua New Guinea | 837.5 (461.0, 1386.0) | 958.7 (544.5, 1563.0) | 269.3 (191.6, 367.9) |
| Philippines | 713.6 (505.0, 990.7) | 900.7 (636.1, 1240.0) | 727.0 (386.0, 1223.0) |
| Republic of Korea | 237.1 (157.9, 345.6) | 316.5 (217.7, 452.2) | 581.7 (413.3, 814.6) |
| Samoa | 765.7 (461.0, 1198.0) | 817.1 (508.4, 1250.0) | 174.6 (112.2, 260.4) |
| Singapore | 234.8 (164.7, 329.4) | 288.2 (208.2, 393.6) | 718.5 (419.4, 1147.0) |
| Solomon Islands | 909.1 (564.1, 1392.0) | 1036.0 (652.4, 1570.0) | 185.6 (125.3, 269.2) |
| Tonga | 624.8 (373.8, 985.7) | 775.7 (471.8, 1206.0) | 785.4 (478.7, 1219.0) |
| Vanuatu | 986.3 (570.4, 1602.0) | 1125.0 (669.3, 1798.0) | 494.1 (289.5, 793.2) |
| Viet Nam | 443.8 (323.9, 593.2) | 776.4 (453.4, 1205.0) | 841.8 (467.0, 1398.0) |

**Table S1B. Overall and compositional NCD crude mortality (per 100 000 population) by sex in the Western Pacific region and 5 select countries, 2000-2019.**

| **Country/area** | **2000** | **2001** | **2002** | **2003** | **2004** | **2005** | **2006** | **2007** | **2008** | **2009** | **2010** | **2011** | **2012** | **2013** | **2014** | **2015** | **2016** | **2017** | **2018** | **2019** |
| --- | --- | --- | --- | --- | --- | --- | --- | --- | --- | --- | --- | --- | --- | --- | --- | --- | --- | --- | --- | --- |
| **Western Pacific region** | | | | | | | | | | | | | | | | | | | | |
| NCD | | | | | | | | | | | | | | | | | | | | |
| Total | 531.5 | 540.1 | 549.7 | 557.5 | 566.7 | 564.1 | 557.6 | 556.1 | 561.7 | 564.7 | 572.3 | 579.7 | 586.3 | 587.5 | 583.4 | 584.9 | 596.9 | 608.7 | 621.0 | 628.5 |
| Male | 582.7 | 593.3 | 603.6 | 612.8 | 626.1 | 626.1 | 618.4 | 619.2 | 629.5 | 636.6 | 645.5 | 656.6 | 665.1 | 667.1 | 665.9 | 665.6 | 680.7 | 690.2 | 697.2 | 707.9 |
| Female | 478.7 | 485.2 | 494.1 | 500.5 | 505.3 | 500.2 | 494.8 | 490.8 | 491.7 | 491.8 | 495.7 | 502.8 | 500.2 | 500.7 | 501.3 | 501.5 | 513.9 | 527.9 | 540.4 | 550.6 |
| Malignant neoplasms | | | | | | | | | | | | | | | | | | | | |
| Total | 142.6 | 144.4 | 146.5 | 149.0 | 152.8 | 153.9 | 153.1 | 153.7 | 155.4 | 156.5 | 158.9 | 160.2 | 160.9 | 161.4 | 162.4 | 163.5 | 166.5 | 169.4 | 172.9 | 176.9 |
| Male | 174.5 | 176.8 | 179.1 | 182.1 | 187.4 | 189.2 | 188.2 | 189.5 | 192.6 | 194.5 | 197.9 | 199.8 | 201.8 | 202.5 | 203.2 | 204.5 | 208.0 | 210.8 | 214.0 | 218.7 |
| Female | 109.8 | 111.1 | 112.8 | 114.8 | 117.2 | 117.4 | 116.8 | 116.7 | 117.1 | 117.3 | 118.7 | 119.3 | 118.7 | 119.0 | 120.2 | 121.2 | 123.7 | 126.7 | 130.5 | 133.9 |
| Diabetes mellitus | | | | | | | | | | | | | | | | | | | | |
| Total | 13.1 | 13.6 | 14.2 | 14.7 | 15.2 | 15.6 | 15.5 | 15.4 | 15.6 | 15.6 | 15.9 | 16.2 | 16.7 | 16.9 | 17.3 | 17.6 | 18.3 | 18.8 | 19.4 | 19.9 |
| Male | 11.8 | 12.3 | 12.9 | 13.4 | 14.0 | 14.4 | 14.4 | 14.4 | 14.7 | 14.8 | 15.1 | 15.5 | 16.2 | 16.4 | 16.8 | 17.1 | 17.8 | 18.3 | 18.7 | 19.2 |
| Female | 14.5 | 15.0 | 15.6 | 16.1 | 16.5 | 16.8 | 16.7 | 16.5 | 16.5 | 16.5 | 16.6 | 16.9 | 17.2 | 17.4 | 17.8 | 18.1 | 18.8 | 19.4 | 20.0 | 20.6 |
| Cardiovascular diseases | | | | | | | | | | | | | | | | | | | | |
| Total | 221.0 | 227.1 | 234.0 | 239.8 | 245.1 | 245.0 | 243.0 | 243.7 | 248.3 | 252.5 | 257.5 | 264.4 | 266.5 | 268.5 | 268.3 | 267.8 | 274.8 | 279.7 | 282.8 | 286.4 |
| Male | 230.1 | 237.2 | 244.2 | 250.4 | 257.5 | 258.8 | 256.6 | 258.9 | 266.0 | 272.4 | 278.8 | 287.1 | 292.8 | 295.5 | 295.3 | 295.0 | 302.6 | 306.2 | 307.9 | 311.4 |
| Female | 211.6 | 216.8 | 223.5 | 228.9 | 232.2 | 230.8 | 229.1 | 228.1 | 230.0 | 231.9 | 235.4 | 241.0 | 239.4 | 240.6 | 240.4 | 239.8 | 246.1 | 252.4 | 257.0 | 260.6 |
| Respiratory diseases | | | | | | | | | | | | | | | | | | | | |
| Total | 90.7 | 90.0 | 89.2 | 87.7 | 86.3 | 82.8 | 78.9 | 76.2 | 74.8 | 73.2 | 71.6 | 71.2 | 69.9 | 68.1 | 66.3 | 64.8 | 65.9 | 66.2 | 67.1 | 67.9 |
| Male | 98.2 | 98.0 | 97.4 | 95.9 | 95.0 | 91.8 | 87.5 | 84.9 | 84.2 | 83.1 | 81.8 | 81.7 | 81.1 | 79.4 | 77.3 | 75.6 | 77.1 | 77.4 | 78.0 | 78.8 |
| Female | 83.0 | 81.6 | 80.7 | 79.2 | 77.3 | 73.5 | 69.9 | 67.1 | 65.0 | 63.0 | 61.2 | 60.3 | 58.2 | 56.5 | 55.0 | 53.7 | 54.3 | 54.6 | 55.9 | 56.7 |
| Others | | | | | | | | | | | | | | | | | | | | |
| Total | 64.1 | 65.0 | 65.8 | 66.3 | 67.2 | 66.9 | 67.1 | 67.1 | 67.6 | 66.8 | 68.5 | 67.7 | 72.3 | 72.6 | 69.1 | 71.0 | 71.5 | 74.6 | 78.8 | 77.3 |
| Male | 68.1 | 69.0 | 70.0 | 71.0 | 72.1 | 71.9 | 71.8 | 71.5 | 72.0 | 71.8 | 71.9 | 72.6 | 73.2 | 73.3 | 73.4 | 73.4 | 75.2 | 77.6 | 78.6 | 79.8 |
| Female | 60.0 | 60.8 | 61.4 | 61.5 | 62.1 | 61.7 | 62.3 | 62.4 | 63.1 | 63.1 | 63.8 | 65.3 | 66.5 | 67.2 | 67.8 | 68.7 | 71.1 | 74.7 | 76.9 | 78.8 |
| **China** | | | | | | | | | | | | | | | | | | | | |
| NCD | | | | | | | | | | | | | | | | | | | | |
| Total | 556.0 | 566.1 | 577.0 | 586.6 | 597.0 | 591.1 | 580.5 | 576.4 | 580.9 | 585.6 | 590.7 | 599.8 | 600.0 | 600.4 | 599.3 | 595.5 | 610.9 | 620.9 | 630.6 | 643.4 |
| Male | 611.8 | 624.5 | 636.4 | 647.5 | 663.7 | 661.2 | 649.2 | 648.1 | 658.3 | 667.4 | 676.3 | 688.2 | 696.2 | 697.9 | 695.7 | 692.3 | 709.4 | 716.5 | 723.0 | 735.7 |
| Female | 497.6 | 505.0 | 514.7 | 522.8 | 527.1 | 517.7 | 508.6 | 501.3 | 499.6 | 499.7 | 501.0 | 507.0 | 499.1 | 498.1 | 498.3 | 494.2 | 507.7 | 520.8 | 534.0 | 547.0 |
| Malignant neoplasms | | | | | | | | | | | | | | | | | | | | |
| Total | 145.2 | 147.4 | 149.7 | 152.8 | 157.1 | 158.0 | 156.5 | 156.7 | 158.3 | 159.7 | 161.9 | 163.3 | 163.8 | 163.8 | 165.0 | 166.0 | 169.6 | 173.2 | 177.6 | 182.9 |
| Male | 178.4 | 181.4 | 184.0 | 187.9 | 193.9 | 195.9 | 194.1 | 195.1 | 198.4 | 201.1 | 204.5 | 206.9 | 209.2 | 209.6 | 210.7 | 212.0 | 216.6 | 220.0 | 224.4 | 230.7 |
| Female | 110.5 | 111.8 | 113.8 | 116.1 | 118.5 | 118.3 | 117.1 | 116.5 | 116.3 | 116.4 | 117.1 | 117.6 | 116.2 | 115.9 | 117.1 | 117.7 | 120.3 | 124.1 | 128.5 | 133.0 |
| Diabetes mellitus | | | | | | | | | | | | | | | | | | | | |
| Total | 11.5 | 11.9 | 12.5 | 13.1 | 13.7 | 13.9 | 13.5 | 13.3 | 13.4 | 13.6 | 13.9 | 14.1 | 14.3 | 14.5 | 14.7 | 14.8 | 15.4 | 15.8 | 16.2 | 16.6 |
| Male | 10.4 | 10.9 | 11.5 | 12.0 | 12.6 | 12.9 | 12.6 | 12.5 | 12.8 | 13.1 | 13.4 | 13.8 | 14.1 | 14.4 | 14.7 | 14.9 | 15.5 | 15.8 | 16.1 | 16.5 |
| Female | 12.6 | 13.0 | 13.6 | 14.2 | 14.8 | 14.9 | 14.4 | 14.1 | 14.1 | 14.1 | 14.3 | 14.5 | 14.4 | 14.5 | 14.7 | 14.8 | 15.3 | 15.8 | 16.3 | 16.7 |
| Cardiovascular diseases | | | | | | | | | | | | | | | | | | | | |
| Total | 230.8 | 238.4 | 246.7 | 253.8 | 260.4 | 259.1 | 256.1 | 256.4 | 261.4 | 267.0 | 272.6 | 280.8 | 282.7 | 285.6 | 285.9 | 284.2 | 292.6 | 297.4 | 301.0 | 305.9 |
| Male | 243.0 | 251.4 | 259.9 | 267.4 | 276.6 | 277.0 | 273.7 | 276.0 | 284.3 | 292.5 | 300.2 | 310.0 | 316.7 | 320.7 | 320.6 | 319.3 | 328.4 | 331.5 | 333.1 | 337.6 |
| Female | 218.1 | 224.8 | 232.9 | 239.6 | 243.5 | 240.4 | 237.7 | 235.9 | 237.5 | 240.4 | 243.8 | 250.1 | 247.0 | 248.7 | 249.5 | 247.3 | 255.1 | 261.7 | 267.4 | 272.8 |
| Respiratory diseases | | | | | | | | | | | | | | | | | | | | |
| Total | 108.0 | 106.8 | 105.6 | 103.6 | 101.7 | 96.8 | 91.6 | 87.8 | 85.5 | 83.4 | 80.8 | 79.6 | 77.3 | 74.8 | 72.2 | 69.6 | 70.6 | 70.5 | 70.9 | 72.0 |
| Male | 115.3 | 114.9 | 113.9 | 111.7 | 110.6 | 106.0 | 100.3 | 96.7 | 95.2 | 93.6 | 91.4 | 90.7 | 89.3 | 86.9 | 83.9 | 81.0 | 82.4 | 82.0 | 81.9 | 82.8 |
| Female | 100.3 | 98.4 | 97.0 | 95.1 | 92.4 | 87.1 | 82.5 | 78.4 | 75.4 | 72.7 | 69.7 | 68.0 | 64.7 | 62.2 | 60.1 | 57.7 | 58.2 | 58.5 | 59.4 | 60.7 |
| Others | | | | | | | | | | | | | | | | | | | | |
| Total | 60.5 | 61.5 | 62.4 | 63.2 | 64.1 | 63.3 | 62.7 | 62.2 | 62.1 | 61.8 | 61.6 | 61.9 | 61.9 | 61.7 | 61.5 | 61.0 | 62.7 | 64.1 | 65.0 | 66.0 |
| Male | 64.8 | 65.9 | 67.2 | 68.5 | 70.0 | 69.3 | 68.4 | 67.7 | 67.7 | 67.2 | 66.8 | 66.8 | 66.7 | 66.3 | 65.8 | 65.0 | 66.4 | 67.2 | 67.5 | 68.1 |
| Female | 56.1 | 56.9 | 57.5 | 57.7 | 58.0 | 57.0 | 56.8 | 56.4 | 56.3 | 56.2 | 56.1 | 56.8 | 56.8 | 56.8 | 56.9 | 56.7 | 58.8 | 60.8 | 62.4 | 63.8 |
| **Japan** | | | | | | | | | | | | | | | | | | | | |
| NCD | | | | | | | | | | | | | | | | | | | | |
| Total | 605.2 | 612.7 | 619.1 | 633.8 | 645.7 | 674.7 | 677.2 | 692.8 | 714.3 | 716.2 | 750.3 | 775.1 | 793.7 | 805.9 | 815.0 | 827.9 | 844.8 | 886.5 | 906.0 | 905.1 |
| Male | 664.5 | 670.9 | 678.5 | 694.1 | 704.9 | 735.7 | 735.6 | 751.4 | 772.4 | 774.9 | 806.9 | 827.4 | 842.7 | 850.7 | 860.5 | 870.4 | 886.4 | 930.3 | 946.3 | 950.1 |
| Female | 548.2 | 556.8 | 562.3 | 576.0 | 589.0 | 616.5 | 621.6 | 636.9 | 659.0 | 660.4 | 696.6 | 725.3 | 747.1 | 763.4 | 772.0 | 787.7 | 805.4 | 845.0 | 867.8 | 862.4 |
| Malignant neoplasms | | | | | | | | | | | | | | | | | | | | |
| Total | 237.4 | 241.2 | 243.9 | 247.5 | 256.3 | 261.1 | 264.4 | 270.3 | 276.5 | 278.1 | 287.1 | 291.8 | 296.5 | 302.0 | 306.1 | 310.2 | 314.4 | 317.0 | 319.9 | 318.8 |
| Male | 292.4 | 295.7 | 299.6 | 303.9 | 314.2 | 320.5 | 323.6 | 331.5 | 338.3 | 339.1 | 349.0 | 353.6 | 358.2 | 363.5 | 367.2 | 371.0 | 373.2 | 376.8 | 376.7 | 375.5 |
| Female | 184.6 | 188.9 | 190.6 | 193.5 | 200.9 | 204.4 | 207.9 | 212.1 | 217.6 | 220.1 | 228.3 | 233.2 | 238.0 | 243.6 | 248.2 | 252.6 | 258.6 | 260.4 | 266.1 | 265.2 |
| Diabetes mellitus | | | | | | | | | | | | | | | | | | | | |
| Total | 9.9 | 9.7 | 10.1 | 10.3 | 10.1 | 10.9 | 11.0 | 11.3 | 11.8 | 11.4 | 11.8 | 12.1 | 12.1 | 11.6 | 11.6 | 11.4 | 11.6 | 12.1 | 12.4 | 12.1 |
| Male | 10.5 | 10.3 | 10.7 | 10.8 | 10.8 | 11.6 | 11.8 | 12.1 | 12.5 | 12.2 | 12.6 | 12.8 | 12.7 | 12.2 | 12.2 | 12.1 | 12.3 | 13.2 | 13.5 | 13.4 |
| Female | 9.3 | 9.2 | 9.5 | 9.8 | 9.4 | 10.3 | 10.2 | 10.6 | 11.1 | 10.7 | 11.1 | 11.5 | 11.4 | 11.0 | 10.9 | 10.7 | 10.9 | 11.0 | 11.3 | 10.9 |
| Cardiovascular diseases | | | | | | | | | | | | | | | | | | | | |
| Total | 233.3 | 234.4 | 236.0 | 242.6 | 241.4 | 255.8 | 252.6 | 255.7 | 262.8 | 259.4 | 270.5 | 277.9 | 281.6 | 280.3 | 278.9 | 279.1 | 281.6 | 293.3 | 293.9 | 287.3 |
| Male | 227.2 | 228.7 | 230.6 | 236.5 | 234.0 | 248.5 | 243.5 | 245.2 | 251.7 | 248.7 | 257.6 | 262.1 | 264.4 | 260.1 | 259.8 | 258.6 | 261.3 | 271.1 | 271.4 | 267.3 |
| Female | 239.1 | 240.0 | 241.2 | 248.4 | 248.4 | 262.7 | 261.3 | 265.8 | 273.3 | 269.7 | 282.6 | 293.0 | 297.9 | 299.4 | 297.0 | 298.5 | 300.9 | 314.3 | 315.1 | 306.1 |
| Respiratory diseases | | | | | | | | | | | | | | | | | | | | |
| Total | 45.4 | 46.8 | 47.8 | 50.5 | 52.1 | 56.3 | 55.0 | 57.7 | 61.2 | 63.2 | 69.5 | 74.4 | 78.5 | 81.6 | 84.5 | 88.4 | 91.7 | 95.3 | 102.7 | 102.6 |
| Male | 53.8 | 54.8 | 55.8 | 59.5 | 60.6 | 65.5 | 64.3 | 67.5 | 71.7 | 74.4 | 81.2 | 86.9 | 91.5 | 94.7 | 98.7 | 102.8 | 107.5 | 113.9 | 122.0 | 123.5 |
| Female | 37.4 | 39.1 | 40.2 | 41.9 | 43.9 | 47.5 | 46.2 | 48.3 | 51.3 | 52.6 | 58.5 | 62.5 | 66.2 | 69.1 | 71.1 | 74.7 | 76.7 | 77.7 | 84.4 | 82.9 |
| Others | | | | | | | | | | | | | | | | | | | | |
| Total | 79.2 | 80.5 | 81.2 | 82.9 | 85.9 | 90.6 | 94.2 | 97.7 | 102.1 | 104.0 | 111.4 | 118.8 | 124.9 | 130.4 | 133.9 | 138.8 | 145.6 | 168.8 | 177.2 | 184.3 |
| Male | 80.5 | 81.4 | 81.7 | 83.4 | 85.3 | 89.6 | 92.3 | 95.1 | 98.3 | 100.6 | 106.5 | 112.1 | 115.8 | 120.1 | 122.5 | 125.9 | 132.0 | 155.3 | 162.7 | 170.5 |
| Female | 77.9 | 79.7 | 80.8 | 82.4 | 86.4 | 91.6 | 96.0 | 100.1 | 105.8 | 107.3 | 116.1 | 125.1 | 133.6 | 140.2 | 144.7 | 151.1 | 158.4 | 181.6 | 190.8 | 197.4 |
| **Viet nam** | | | | | | | | | | | | | | | | | | | | |
| NCD | | | | | | | | | | | | | | | | | | | | |
| Total | 472.3 | 480.7 | 488.3 | 493.6 | 501.1 | 507.0 | 517.7 | 530.7 | 542.4 | 550.9 | 556.8 | 566.9 | 575.8 | 581.8 | 587.5 | 592.6 | 601.4 | 608.6 | 614.7 | 618.4 |
| Male | 489.5 | 500.5 | 510.4 | 516.4 | 524.6 | 530.8 | 542.8 | 559.4 | 574.8 | 585.6 | 592.7 | 605.5 | 617.0 | 626.3 | 634.2 | 641.2 | 652.5 | 661.7 | 669.4 | 675.0 |
| Female | 455.7 | 461.8 | 467.1 | 471.7 | 478.4 | 484.1 | 493.3 | 503.0 | 511.1 | 517.3 | 522.1 | 529.5 | 535.8 | 538.6 | 542.0 | 545.3 | 551.6 | 556.9 | 561.4 | 563.3 |
| Malignant neoplasms | | | | | | | | | | | | | | | | | | | | |
| Total | 97.5 | 97.9 | 97.9 | 98.5 | 99.6 | 101.2 | 102.4 | 105.0 | 107.1 | 109.3 | 111.0 | 112.0 | 112.8 | 114.4 | 114.6 | 115.6 | 116.9 | 118.1 | 119.6 | 120.9 |
| Male | 126.8 | 126.8 | 126.3 | 126.4 | 127.3 | 128.8 | 130.2 | 134.2 | 137.1 | 140.1 | 142.2 | 143.4 | 144.4 | 146.8 | 146.3 | 147.2 | 148.5 | 149.7 | 151.1 | 152.6 |
| Female | 69.4 | 70.1 | 70.6 | 71.6 | 72.8 | 74.6 | 75.5 | 76.7 | 78.0 | 79.5 | 80.7 | 81.5 | 82.1 | 82.9 | 83.8 | 84.9 | 86.1 | 87.3 | 88.8 | 89.9 |
| Diabetes mellitus | | | | | | | | | | | | | | | | | | | | |
| Total | 27.4 | 27.9 | 28.4 | 28.8 | 29.3 | 29.8 | 30.6 | 31.6 | 32.6 | 33.5 | 34.3 | 35.7 | 37.3 | 38.6 | 40.2 | 41.7 | 43.3 | 44.6 | 46.0 | 47.2 |
| Male | 17.6 | 18.6 | 19.5 | 20.3 | 21.0 | 21.7 | 22.6 | 23.6 | 24.7 | 25.5 | 26.3 | 27.5 | 28.8 | 29.9 | 31.3 | 32.3 | 33.5 | 34.4 | 35.3 | 36.1 |
| Female | 36.7 | 36.8 | 37.0 | 37.1 | 37.3 | 37.5 | 38.3 | 39.3 | 40.3 | 41.2 | 42.2 | 43.8 | 45.5 | 47.1 | 49.0 | 50.8 | 52.9 | 54.6 | 56.4 | 58.0 |
| Cardiovascular diseases | | | | | | | | | | | | | | | | | | | | |
| Total | 226.0 | 232.6 | 238.8 | 243.0 | 248.3 | 252.7 | 259.9 | 267.4 | 274.2 | 279.0 | 281.5 | 286.0 | 289.6 | 290.8 | 292.5 | 292.8 | 295.5 | 297.6 | 299.2 | 299.9 |
| Male | 221.0 | 229.4 | 237.5 | 242.4 | 248.3 | 252.9 | 261.0 | 270.1 | 279.1 | 284.9 | 288.2 | 294.6 | 300.3 | 303.7 | 308.1 | 310.5 | 315.6 | 319.9 | 323.7 | 326.6 |
| Female | 230.7 | 235.6 | 240.0 | 243.5 | 248.3 | 252.4 | 258.8 | 264.8 | 269.5 | 273.2 | 275.1 | 277.6 | 279.2 | 278.2 | 277.3 | 275.6 | 275.8 | 275.8 | 275.3 | 273.9 |
| Respiratory diseases | | | | | | | | | | | | | | | | | | | | |
| Total | 43.7 | 44.6 | 45.2 | 45.5 | 45.7 | 45.7 | 46.2 | 46.4 | 46.4 | 46.0 | 45.5 | 45.8 | 46.1 | 46.0 | 46.2 | 46.2 | 46.7 | 47.0 | 47.1 | 47.1 |
| Male | 49.8 | 51.2 | 52.4 | 52.9 | 53.2 | 53.2 | 54.0 | 54.5 | 54.9 | 54.6 | 54.1 | 54.7 | 55.3 | 55.5 | 55.9 | 56.0 | 56.8 | 57.3 | 57.5 | 57.6 |
| Female | 37.9 | 38.2 | 38.4 | 38.4 | 38.4 | 38.4 | 38.6 | 38.5 | 38.2 | 37.6 | 37.2 | 37.2 | 37.1 | 36.9 | 36.8 | 36.7 | 36.8 | 36.9 | 36.9 | 36.8 |
| Others | | | | | | | | | | | | | | | | | | | | |
| Total | 77.7 | 77.8 | 78.0 | 77.9 | 78.1 | 77.7 | 78.7 | 80.4 | 82.1 | 83.2 | 84.5 | 87.4 | 90.1 | 92.0 | 93.8 | 96.3 | 99.0 | 101.3 | 102.9 | 103.4 |
| Male | 74.3 | 74.5 | 74.7 | 74.4 | 74.7 | 74.1 | 75.1 | 76.9 | 79.1 | 80.5 | 82.0 | 85.2 | 88.3 | 90.5 | 92.5 | 95.2 | 98.1 | 100.4 | 101.8 | 102.1 |
| Female | 80.9 | 81.0 | 81.1 | 81.2 | 81.4 | 81.1 | 82.2 | 83.7 | 85.0 | 85.8 | 86.9 | 89.4 | 91.8 | 93.6 | 95.1 | 97.3 | 99.9 | 102.2 | 103.9 | 104.7 |
| **Australia** | | | | | | | | | | | | | | | | | | | | |
| NCD | | | | | | | | | | | | | | | | | | | | |
| Total | 59.2 | 585.2 | 59.6 | 580.9 | 58.3 | 594.5 | 60.0 | 591.1 | 61.1 | 585.1 | 58.2 | 597.7 | 58.7 | 580.0 | 59.1 | 601.5 | 59.2 | 588.4 | 57.5 | 578.6 |
| Male | 611.2 | 603.1 | 612.3 | 596.3 | 599.2 | 608.4 | 612.8 | 602.9 | 621.2 | 596.9 | 592.1 | 613.0 | 598.5 | 597.4 | 604.3 | 617.0 | 614.9 | 608.3 | 598.6 | 601.9 |
| Female | 573.8 | 567.7 | 580.4 | 565.7 | 567.6 | 580.8 | 586.5 | 579.4 | 601.1 | 573.4 | 572.4 | 582.5 | 575.8 | 562.7 | 577.6 | 586.2 | 570.0 | 568.9 | 551.6 | 555.7 |
| Malignant neoplasms | | | | | | | | | | | | | | | | | | | | |
| Total | 185.2 | 186.6 | 188.0 | 185.9 | 188.7 | 190.2 | 193.3 | 187.7 | 193.4 | 188.0 | 191.1 | 192.6 | 189.0 | 190.3 | 188.1 | 192.8 | 189.6 | 186.7 | 189.8 | 187.9 |
| Male | 211.2 | 212.1 | 212.7 | 210.2 | 214.6 | 216.9 | 221.1 | 214.6 | 220.5 | 214.5 | 218.5 | 220.8 | 215.9 | 217.9 | 212.9 | 219.2 | 217.0 | 213.4 | 216.7 | 214.2 |
| Female | 159.6 | 161.5 | 163.6 | 162.0 | 163.3 | 163.8 | 165.9 | 161.2 | 166.7 | 161.7 | 163.8 | 164.8 | 162.3 | 163.1 | 163.7 | 166.7 | 162.5 | 160.5 | 163.3 | 162.0 |
| Diabetes mellitus | | | | | | | | | | | | | | | | | | | | |
| Total | 15.4 | 15.6 | 16.4 | 16.8 | 17.6 | 17.5 | 18.3 | 18.1 | 19.5 | 19.1 | 17.7 | 19.0 | 18.9 | 19.0 | 18.8 | 19.9 | 20.1 | 20.1 | 19.0 | 19.6 |
| Male | 16.4 | 16.7 | 17.6 | 18.1 | 18.4 | 18.1 | 18.5 | 18.5 | 20.1 | 19.5 | 18.0 | 19.9 | 19.8 | 20.4 | 19.4 | 21.3 | 22.0 | 21.8 | 21.0 | 21.6 |
| Female | 14.5 | 14.5 | 15.1 | 15.6 | 16.8 | 16.8 | 18.1 | 17.8 | 18.9 | 18.7 | 17.5 | 18.1 | 18.0 | 17.6 | 18.3 | 18.5 | 18.3 | 18.5 | 17.0 | 17.7 |
| Cardiovascular diseases | | | | | | | | | | | | | | | | | | | | |
| Total | 250.5 | 244.9 | 243.6 | 234.5 | 230.2 | 230.1 | 226.0 | 218.7 | 223.7 | 208.1 | 200.7 | 202.9 | 193.3 | 188.4 | 191.8 | 190.4 | 182.8 | 178.2 | 168.5 | 166.6 |
| Male | 239.9 | 235.8 | 234.1 | 225.9 | 222.7 | 220.1 | 216.2 | 209.1 | 212.1 | 199.3 | 191.5 | 196.9 | 186.7 | 184.7 | 186.7 | 186.7 | 182.9 | 177.5 | 170.0 | 168.9 |
| Female | 260.9 | 253.8 | 252.9 | 242.9 | 237.6 | 240.0 | 235.8 | 228.3 | 235.2 | 216.9 | 209.8 | 209.0 | 199.9 | 192.1 | 196.8 | 194.2 | 182.8 | 178.9 | 166.9 | 164.4 |
| Respiratory diseases | | | | | | | | | | | | | | | | | | | | |
| Total | 44.6 | 43.1 | 46.1 | 43.3 | 43.2 | 45.2 | 43.1 | 45.6 | 44.7 | 44.8 | 45.8 | 48.1 | 49.1 | 45.5 | 48.7 | 49.4 | 49.6 | 50.6 | 47.8 | 49.8 |
| Male | 51.0 | 48.7 | 51.2 | 48.2 | 47.2 | 49.9 | 47.5 | 49.6 | 48.6 | 49.1 | 49.5 | 52.3 | 53.6 | 50.2 | 53.2 | 52.7 | 53.3 | 53.9 | 51.4 | 52.5 |
| Female | 38.3 | 37.6 | 41.0 | 38.5 | 39.2 | 40.5 | 38.8 | 41.7 | 40.9 | 40.5 | 42.2 | 43.9 | 44.6 | 40.8 | 44.3 | 46.2 | 45.9 | 47.2 | 44.3 | 47.0 |
| Others | | | | | | | | | | | | | | | | | | | | |
| Total | 96.6 | 95.1 | 102.2 | 100.3 | 103.7 | 111.6 | 118.8 | 120.9 | 129.7 | 125.0 | 126.9 | 135.0 | 136.9 | 136.8 | 143.4 | 148.9 | 150.2 | 152.8 | 149.9 | 154.7 |
| Male | 92.6 | 89.8 | 96.6 | 93.9 | 96.5 | 103.4 | 109.5 | 111.2 | 119.9 | 114.4 | 114.6 | 123.1 | 122.5 | 124.2 | 132.1 | 137.1 | 139.7 | 141.7 | 139.6 | 144.7 |
| Female | 100.5 | 100.3 | 107.8 | 106.7 | 110.8 | 119.7 | 128.0 | 130.4 | 139.4 | 135.6 | 139.1 | 146.7 | 151.1 | 149.1 | 154.6 | 160.5 | 160.5 | 163.8 | 160.0 | 164.6 |
| **Papua new guinea** | | | | | | | | | | | | | | | | | | | | |
| NCD | | | | | | | | | | | | | | | | | | | | |
| Total | 345.5 | 359.5 | 356.2 | 367.7 | 372.9 | 374.1 | 378.2 | 387.6 | 384.5 | 376.5 | 383.8 | 383.9 | 393.9 | 391.2 | 394.0 | 391.4 | 391.9 | 390.3 | 392.4 | 395.7 |
| Male | 363.3 | 377.9 | 372.7 | 383.5 | 387.8 | 388.4 | 392.0 | 401.4 | 397.9 | 388.6 | 396.2 | 398.0 | 410.5 | 408.8 | 414.9 | 415.4 | 416.6 | 415.5 | 419.6 | 426.1 |
| Female | 326.1 | 339.5 | 338.1 | 350.5 | 356.7 | 358.6 | 363.1 | 372.7 | 370.0 | 363.3 | 370.4 | 368.7 | 375.9 | 372.1 | 371.5 | 365.5 | 365.3 | 363.2 | 363.1 | 363.0 |
| Malignant neoplasms | | | | | | | | | | | | | | | | | | | | |
| Total | 73.7 | 73.5 | 73.6 | 73.3 | 73.1 | 73.4 | 73.7 | 74.1 | 74.1 | 74.0 | 75.2 | 75.9 | 75.7 | 75.0 | 74.9 | 75.4 | 75.5 | 76.6 | 77.8 | 78.2 |
| Male | 66.7 | 66.7 | 66.8 | 66.3 | 65.9 | 66.1 | 66.5 | 66.8 | 66.8 | 66.9 | 67.7 | 68.6 | 68.8 | 68.5 | 68.5 | 69.1 | 69.8 | 71.1 | 72.5 | 73.1 |
| Female | 81.3 | 81.0 | 81.0 | 80.9 | 80.9 | 81.3 | 81.6 | 82.0 | 81.9 | 81.7 | 83.4 | 83.7 | 83.3 | 82.1 | 81.7 | 82.1 | 81.8 | 82.6 | 83.5 | 83.7 |
| Diabetes mellitus | | | | | | | | | | | | | | | | | | | | |
| Total | 37.3 | 39.7 | 39.7 | 41.8 | 43.4 | 44.1 | 44.3 | 45.5 | 44.9 | 43.6 | 44.6 | 44.7 | 46.9 | 47.1 | 48.3 | 48.7 | 49.4 | 49.0 | 49.7 | 50.8 |
| Male | 46.5 | 49.4 | 49.2 | 51.4 | 53.0 | 53.5 | 53.6 | 54.9 | 54.1 | 52.6 | 53.5 | 53.8 | 56.2 | 56.3 | 57.7 | 58.3 | 58.9 | 58.8 | 59.9 | 61.4 |
| Female | 27.4 | 29.2 | 29.5 | 31.3 | 33.0 | 33.8 | 34.1 | 35.3 | 34.8 | 33.9 | 34.9 | 34.9 | 36.8 | 37.1 | 38.0 | 38.3 | 39.1 | 38.6 | 38.9 | 39.6 |
| Cardiovascular diseases | | | | | | | | | | | | | | | | | | | | |
| Total | 122.5 | 130.2 | 129.5 | 135.8 | 139.5 | 140.5 | 143.2 | 148.5 | 147.7 | 144.5 | 148.1 | 148.0 | 153.6 | 152.9 | 154.8 | 153.6 | 154.0 | 152.4 | 153.2 | 155.1 |
| Male | 137.8 | 146.1 | 144.3 | 150.6 | 154.0 | 154.7 | 157.2 | 162.9 | 161.8 | 158.1 | 162.2 | 162.9 | 169.9 | 169.6 | 173.1 | 173.3 | 173.7 | 172.6 | 174.2 | 177.5 |
| Female | 105.7 | 112.9 | 113.4 | 119.7 | 123.7 | 125.1 | 127.9 | 133.0 | 132.3 | 129.8 | 132.8 | 131.9 | 135.8 | 134.8 | 135.0 | 132.3 | 132.7 | 130.8 | 130.6 | 131.2 |
| Respiratory diseases | | | | | | | | | | | | | | | | | | | | |
| Total | 70.0 | 72.8 | 71.8 | 73.9 | 74.5 | 73.8 | 74.7 | 76.2 | 75.5 | 73.9 | 74.5 | 74.0 | 75.4 | 74.4 | 73.8 | 72.0 | 71.8 | 71.2 | 70.9 | 71.1 |
| Male | 63.4 | 65.7 | 64.6 | 65.8 | 65.8 | 65.0 | 65.6 | 66.7 | 66.1 | 64.5 | 64.9 | 64.8 | 66.1 | 65.4 | 65.4 | 64.4 | 64.4 | 64.2 | 64.4 | 64.8 |
| Female | 77.1 | 80.6 | 79.7 | 82.8 | 83.9 | 83.4 | 84.5 | 86.5 | 85.7 | 84.1 | 85.0 | 84.0 | 85.4 | 84.1 | 82.8 | 80.1 | 79.9 | 78.7 | 78.0 | 77.9 |
| Others | | | | | | | | | | | | | | | | | | | | |
| Total | 42.1 | 43.2 | 41.5 | 42.9 | 42.4 | 42.3 | 42.4 | 43.3 | 42.4 | 40.5 | 41.4 | 41.3 | 42.3 | 41.8 | 42.4 | 41.8 | 41.1 | 41.0 | 40.7 | 40.4 |
| Male | 48.8 | 50.1 | 47.8 | 49.4 | 49.0 | 49.1 | 49.1 | 50.2 | 49.1 | 46.6 | 48.0 | 47.9 | 49.6 | 49.0 | 50.2 | 50.3 | 49.8 | 48.9 | 48.7 | 49.4 |
| Female | 34.7 | 35.8 | 34.6 | 35.8 | 35.2 | 35.0 | 35.1 | 35.9 | 35.2 | 33.7 | 34.3 | 34.1 | 34.5 | 34.0 | 34.0 | 32.6 | 31.8 | 32.6 | 32.1 | 30.7 |

Data were obtained from the WHO-GLOBAL HEALTH OBSERVATORY: https://www.who.int/data/gho

**Table S1C. Overall age-standardized NCD mortality (per 100 000 population) by sex in the Western Pacific region and 5 selected countries, 2000-2019.**

| **Country/area** | **2000** | **2001** | **2002** | **2003** | **2004** | **2005** | **2006** | **2007** | **2008** | **2009** | **2010** | **2011** | **2012** | **2013** | **2014** | **2015** | **2016** | **2017** | **2018** | **2019** |
| --- | --- | --- | --- | --- | --- | --- | --- | --- | --- | --- | --- | --- | --- | --- | --- | --- | --- | --- | --- | --- |
| **Western Pacific region** | | | | | | | | | | | | | | | | | | | | |
| Total | 601.1 (473.5, 743.6) | 595.3 (471.6, 735.4) | 591.4 (472.4, 727.1) | 586.1 (470.1, 717.0) | 583.8 (470.5, 713.8) | 571.6 (462.4, 698.2) | 548.5 (444.4, 669.1) | 532.6 (431.0, 648.9) | 525.7 (425.0, 641.9) | 518.2 (418.7, 634.4) | 514.3 (413.1, 633.2) | 506.4 (404.4, 626.9) | 495.1 (394.3, 615.4) | 483.9 (382.3, 605.8) | 473.0 (370.0, 596.2) | 463.5 (357.2, 591.0) | 458.9 (349.5, 591.8) | 454.0 (340.2, 591.8) | 448.3 (332.3, 593.8) | 443.8 (323.9, 593.2) |
| Male | 750.1 (605.9, 908.9) | 744.4 (604.3, 902.4) | 738.4 (604.8, 891.0) | 731.2 (601.6, 878.2) | 732.6 (605.7, 879.5) | 722.6 (599.9, 867.3) | 692.4 (575.2, 829.6) | 675.5 (560.0, 808.0) | 672.0 (556.5, 804.9) | 667.2 (552.1, 800.5) | 665.8 (548.2, 801.7) | 656.6 (538.6, 794.3) | 647.2 (529.9, 785.5) | 633.6 (514.1, 774.7) | 619.2 (495.6, 761.7) | 608.2 (481.1, 756.5) | 601.1 (470.0, 755.7) | 591.1 (454.5, 750.5) | 580.3 (441.3, 748.6) | 574.1 (430.0, 749.2) |
| Female | 480.6 (368.8, 607.6) | 474.3 (366.2, 597.3) | 471.9 (367.1, 591.5) | 467.7 (365.2, 583.2) | 462.7 (362.7, 576.3) | 449.8 (353.8, 559.2) | 431.6 (340.4, 536.2) | 416.4 (328.3, 517.2) | 406.9 (320.5, 507.1) | 397.9 (313.1, 497.8) | 392.6 (306.8, 495.5) | 385.4 (298.3, 489.4) | 372.5 (287.2, 475.7) | 363.1 (278.1, 466.9) | 354.9 (270.6, 459.7) | 347.2 (259.9, 455.2) | 343.9 (254.2, 456.3) | 342.2 (249.3, 459.6) | 340.0 (244.9, 463.7) | 337.0 (239.0, 462.4) |
| **China** | | | | | | | | | | | | | | | | | | | | |
| Total | 687.6 (552.1, 831.4) | 682.4 (551.9, 823.7) | 678.4 (554.4, 814.3) | 673.7 (553.5, 803.7) | 673.4 (556.8, 802.5) | 659.1 (548.0, 783.7) | 628.0 (523.9, 744.6) | 607.4 (506.7, 718.0) | 599.1 (500.3, 709.2) | 593.9 (496.3, 704.6) | 590.7 (491.2, 704.6) | 579.7 (478.9, 695.5) | 562.7 (464.2, 677.4) | 548.6 (449.2, 665.2) | 535.3 (434.3, 653.2) | 521.4 (416.5, 643.8) | 514.5 (406.1, 643.3) | 505.1 (392.2, 638.5) | 496.6 (381.2, 639.4) | 491.5 (370.8, 638.9) |
| Male | 837.3 (687.4, 994.4) | 832.4 (687.6, 989.4) | 825.7 (689.6, 976.1) | 818.2 (687.5, 961.6) | 823.6 (696.3, 967.0) | 813.0 (691.3, 953.8) | 774.2 (659.7, 905.4) | 753.7 (641.3, 878.6) | 750.7 (639.0, 875.8) | 751.1 (639.6, 876.8) | 753.2 (638.6, 881.4) | 742.0 (626.6, 872.7) | 730.0 (616.2, 860.9) | 714.7 (597.5, 848.8) | 697.9 (575.9, 833.3) | 683.9 (558.0, 825.6) | 674.0 (543.5, 822.7) | 657.2 (521.1, 809.7) | 642.2 (503.2, 805.4) | 635.6 (490.2, 806.9) |
| Female | 561.2 (440.6, 691.4) | 555.0 (439.1, 680.5) | 553.0 (441.6, 674.2) | 550.1 (441.4, 666.3) | 545.1 (439.9, 659.4) | 529.0 (429.2, 637.4) | 503.8 (410.8, 605.6) | 483.3 (394.7, 579.6) | 470.9 (385.1, 566.3) | 462.2 (378.2, 557.9) | 455.9 (371.0, 555.4) | 444.9 (358.2, 545.8) | 424.3 (340.7, 523.1) | 411.7 (328.7, 511.1) | 401.6 (319.9, 502.2) | 389.2 (303.9, 493.2) | 384.0 (295.9, 493.6) | 379.8 (288.3, 494.4) | 376.0 (282.1, 498.8) | 372.7 (274.5, 497.2) |
| **Japan** | | | | | | | | | | | | | | | | | | | | |
| Total | 303.0 (247.0, 367.8) | 295.5 (239.8, 360.0) | 288.3 (232.9, 352.6) | 284.8 (229.3, 349.4) | 280.7 (225.4, 345) | 282.1 (225.6, 347.7) | 273.1 (217.5, 337.9) | 269.8 (213.3, 335.5) | 268.4 (210.3, 335.8) | 260.3 (202.7, 327.2) | 262.1 (202.9, 330.5) | 260.5 (201.3, 328.8) | 257.1 (197.8, 325.7) | 252.5 (193.5, 320.9) | 247.8 (189.2, 315.7) | 243.1 (184.7, 311.2) | 240.4 (182.6, 308.0) | 242.8 (183.5, 312.5) | 240.8 (181.5, 310.6) | 235.4 (177.3, 304.2) |
| Male | 411.7 (349.3, 484.8) | 400.7 (338.9, 473.2) | 391.4 (329.8, 463.8) | 387.4 (325.6, 459.8) | 381.0 (319.3, 453.4) | 384.6 (321.2, 458.9) | 370.5 (308.4, 443.1) | 365.8 (302.9, 439.5) | 363.4 (298.8, 439.1) | 353.4 (289.0, 428.8) | 355.5 (289.3, 432.9) | 350.3 (284.6, 426.9) | 344.1 (278.7, 420.6) | 336.3 (271.4, 412.3) | 329.8 (265.1, 405.8) | 323.4 (258.9, 399.6) | 318.3 (254.4, 393.9) | 322.0 (255.8, 400.5) | 317.6 (251.8, 396.1) | 310.7 (245.6, 389.0) |
| Female | 223.2 (172.7, 281.2) | 217.3 (166.9, 275.3) | 211.4 (161.4, 269.1) | 207.9 (157.8, 265.9) | 205.3 (155.4, 262.9) | 205.6 (154.9, 264.2) | 199.2 (149.2, 257.3) | 196.3 (145.4, 255.3) | 195.4 (143.1, 255.9) | 188.6 (136.7, 248.6) | 190.4 (137.2, 251.8) | 190.7 (137.0, 252.2) | 188.7 (134.8, 250.6) | 186.1 (132.4, 247.9) | 182.8 (129.7, 243.9) | 179.4 (126.4, 240.6) | 178.1 (125.8, 238.8) | 179.2 (125.9, 241.2) | 178.7 (125.3, 240.9) | 174.3 (122.6, 235.1) |
| **Viet Nam** | | | | | | | | | | | | | | | | | | | | |
| Total | 592.6 (354.3, 916.9) | 590.2 (355.6, 906.6) | 589.5 (358.0, 899.2) | 587.2 (359.3, 889.5) | 588.5 (363.3, 885.8) | 587.9 (366.4, 878.4) | 589.0 (370.2, 874.1) | 593.2 (374.8, 875.7) | 596.1 (377.8, 878.4) | 596.5 (377.0, 878.5) | 595.2 (375.0, 879.8) | 595.3 (372.5, 885.4) | 594.8 (368.2, 891.4) | 592.2 (362.7, 894.9) | 589.6 (357.5, 898.7) | 587.0 (351.8, 903.2) | 583.6 (345.4, 903.9) | 579.5 (339.2, 905.0) | 575.2 (333.3, 904.8) | 569.4 (326.9, 900.9) |
| Male | 781.7 (470.0, 1186.0) | 782.9 (474.6, 1180.0) | 785.9 (480.4, 1177.0) | 784.8 (483.6, 1167.0) | 788.7 (489.9, 1165.0) | 790.1 (495.9, 1159.0) | 792.3 (501.1, 1155.0) | 800.2 (509.2, 1161.0) | 806.5 (514.8, 1169.0) | 808.6 (514.8, 1173.0) | 807.7 (512.4, 1176.0) | 807.9 (509.8, 1183.0) | 807.6 (504.2, 1192) | 805.8 (497.9, 1197.0) | 803.6 (491.9, 1204.0) | 800.9 (485.4, 1210) | 796.3 (478.1, 1211) | 790.5 (470.1, 1213.0) | 784.2 (462.0, 1211.0) | 776.4 (453.4, 1205.0) |
| Female | 457.4 (272.4, 719.3) | 452.2 (271.2, 706.0) | 448.9 (271.3, 696.0) | 446.0 (271.4, 687.4) | 445.6 (273.7, 682.3) | 443.9 (274.9, 674.6) | 443.9 (277.3, 669.9) | 444.8 (279.3, 667.6) | 444.6 (279.9, 665.9) | 443.6 (278.4, 663.2) | 441.9 (276.7, 663.1) | 441.1 (273.7, 666.0) | 440.1 (270.2, 669.7) | 436.6 (265.2, 670.8) | 433.8 (260.7, 672.3) | 431.4 (255.7, 675.0) | 428.3 (249.9, 675.0) | 425.1 (244.9, 675.4) | 422.1 (240.6, 675.9) | 417.6 (235.8, 673.1) |
| **Australia** | | | | | | | | | | | | | | | | | | | | |
| Total | 386.1 (311.8, 475.8) | 373.4 (300.4, 461.9) | 372.1 (298.0, 462.7) | 357.4 (284.8, 447.1) | 351.3 (278.2, 440.5) | 349.8 (275.3, 440.9) | 343.2 (268.9, 434.3) | 333.8 (261.0, 422.8) | 338.1 (263.3, 429.9) | 321.8 (249.6, 410.5) | 315.7 (244.0, 404.6) | 315.6 (242.9, 406.2) | 304.4 (232.7, 394.7) | 298.8 (226.9, 389.9) | 300.5 (226.2, 394.9) | 302.2 (224.9, 401.3) | 293.1 (215.7, 393.3) | 287.3 (208.7, 389.7) | 278.9 (200.4, 381.2) | 278.5 (197.5, 385.1) |
| Male | 480.3 (398.3, 580.7) | 462.6 (382.1, 562.0) | 458.8 (377.2, 560.6) | 438.8 (359.1, 540.0) | 431.3 (351.1, 531.4) | 428.6 (347.2, 530.1) | 418.6 (337.9, 520.0) | 403.6 (325.3, 501.9) | 407.9 (327.6, 508.8) | 387.5 (310.1, 485.5) | 378.7 (302.3, 476.4) | 379.7 (301.7, 480.2) | 362.6 (286.5, 462.5) | 357.2 (280.2, 459.1) | 355.6 (276.8, 460.6) | 358.1 (275.5, 468.3) | 348.9 (265.2, 461.5) | 339.5 (254.7, 453.7) | 330.2 (244.3, 445.1) | 328.4 (239.4, 447.9) |
| Female | 310.2 (243.7, 389.9) | 301.0 (235.6, 379.4) | 301.9 (235.3, 382.1) | 290.0 (224.7, 368.9) | 285.1 (219.4, 364.3) | 284.3 (217.1, 365.3) | 280.2 (212.8, 361.5) | 273.9 (207.5, 353.7) | 278.0 (209.4, 360.6) | 264.6 (198.5, 344.1) | 260.6 (194.5, 340.6) | 259.9 (193.0, 340.8) | 253.3 (186.8, 334.3) | 247.2 (180.9, 328.0) | 251.3 (182.3, 335.5) | 252.1 (180.8, 340.3) | 243.1 (172.4, 331.5) | 240.2 (168.3, 331.3) | 232.5 (161.5, 322.9) | 233.5 (160.2, 327.7) |
| **Papua New Guinea** | | | | | | | | | | | | | | | | | | | | |
| Total | 750.3 (399.5, 1265) | 773.8 (412.3, 1305) | 773.1 (412.4, 1302) | 795.6 (426.5, 1337) | 811.8 (437.2, 1359) | 820.1 (444.5, 1372) | 824.2 (447.5, 1378.0) | 835.2 (453.6, 1394.0) | 829.1 (450.9, 1385.0) | 817.6 (444.8, 1365.0) | 828.7 (452.0, 1382.0) | 828.5 (452.9, 1380.0) | 843.9 (461.6, 1407.0) | 840.8 (459.4, 1400.0) | 846.5 (462.4, 1410.0) | 847.5 (463.8, 1412.0) | 842.7 (461.6, 1401.0) | 835.7 (457.9, 1387.0) | 835.5 (458.5, 1384.0) | 837.5 (461.0, 1386.0) |
| Male | 896.1 (495.8, 1485) | 923.3 (511.3, 1529) | 919.3 (510.2, 1517) | 940.9 (524.6, 1549) | 954.9 (534.3, 1566) | 961.3 (541, 1575.0) | 964.2 (543.0, 1580.0) | 975.5 (549.3, 1597.0) | 967.6 (546.4, 1587.0) | 952.3 (537.9, 1561.0) | 963.3 (544.8, 1578.0) | 961.5 (544.9, 1578.0) | 977.4 (553.8, 1607.0) | 970.6 (549.9, 1592.0) | 976.1 (551.5, 1602.0) | 975.2 (552.3, 1602.0) | 966.5 (547.1, 1585.0) | 957.0 (542.2, 1566.0) | 956.6 (541.9, 1562.0) | 958.7 (544.5, 1563.0) |
| Female | 632.6 (324.2, 1085) | 652.8 (334.6, 1120) | 655.1 (335.9, 1124) | 677.9 (349.6, 1162) | 695.7 (360.9, 1187) | 705.3 (368.5, 1202) | 710.3 (372.2, 1209.0) | 721.0 (378.1, 1224.0) | 716.1 (375.5, 1216.0) | 707.6 (371.1, 1201.0) | 718.6 (378.4, 1217.0) | 718.5 (379, 1213.0) | 732.1 (386.6, 1236.0) | 730.9 (384.9, 1235.0) | 735.1 (387.6, 1243.0) | 735.8 (388.1, 1242.0) | 733.2 (387.7, 1237.0) | 727.6 (384.3, 1226.0) | 726.6 (384.5, 1223.0) | 727.0 (386.0, 1223.0) |

Data was obtained from the WHO-GLOBAL HEALTH OBSERVATORY: https://www.who.int/data/gho

**Table S2. Prevalence and management rates (%) of hypertension in countries of the Western Pacific region based on previous research.**

| First author | Publish | Project | Survey year | Areas | Sample size | Age range | Hypertension definition | Prevalence | | | Awareness | | | Treatment | | | Control | | |
| --- | --- | --- | --- | --- | --- | --- | --- | --- | --- | --- | --- | --- | --- | --- | --- | --- | --- | --- | --- |
|  |  |  |  |  |  |  |  | Total | Male | Female | Total | Male | Female | Total | Male | Female | Total | Male | Female |
|  |  |  |  |  |  |  |  | % | % | % | % | % | % | % | % | % | % | % | % |
| Okada, A^1^ | 2022 | DeSC database | 2019-2020 | Japan | 1,932,021 | All age | - | 20.9 | - | - | - | - | - | - | - | - | - | - | - |
| Tan, M^2^ | 2022 | SEACO HDSS database | 2013-2014 | Malaysia | 18101 | 18–97 | - | 18.4 | - | - | - | - | - | - | - | - | - | - | - |
| An, S^3^ | 2022 | KNHANES 2007–2014 | 2007-2014 | Republic of Korea | 26492 | 40–69 | - | 49.9 | 58.7 | 41.0 | - | - | - | - | - | - | - | - | - |
| Tafuna'i, M^4^ | 2022 | - | 2019 | Samoa | 1163 | ≥15 | BP≥140/90 mmHg#* | 54.3 | - | - | - | - | - | - | - | - | - | - | - |
| LaMonica, L^5^ | 2022 | - | 2018 | Samoa | 689 | 29.5–50.9 | BP≥140/90 mmHg* | 31.0 | 35.8 | 26.6 | 11.8 | 7.6 | 17.2 | 9.4 | - | - | 2.8 | - | - |
| Kirschbaum, T^6^ | 2021 | STEPS | 2010 | Cambodia | 5314 | 25–64 | BP≥140/90 mmHg* | 12.6 | - | - | - | - | - | - | - | - | - | - | - |
|  |  | CHNS | 2009 | China | 9741 | 15–99 | BP≥140/90 mmHg* | 29.1 | - | - | - | - | - | - | - | - | - | - | - |
|  |  | STEPS | 2009 | Mongolia | 5409 | 15–64 | BP≥140/90 mmHg* | 31.6 | - | - | - | - | - | - | - | - | - | - | - |
|  |  | STEPS | 2011 | Vanuatu | 4515 | 25–64 | BP≥140/90 mmHg* | 29.9 | - | - | - | - | - | - | - | - | - | - | - |
| Yu, E^7^ | 2021 | Hong Kong Population Health Survey | 2014-2015 | Hong Kong SAR | 1551 | 18–64 | SBP≥130 mmHg;  DBP≥80 mmHg | 12.5;  33.2 | - | - | - | - | - | - | - | - | - | - | - |
| Liew, S^8^ | 2019 | MEC | 2004-2010 | Singapore | 10215 | ≥21 | BP≥140/90 mmHg# | 31.1 | 33.9 | 29.0 | 51.0 | - | - | 74.8 | - | - | - | - | - |
| Wang, Z^9^ | 2018 | China Hypertension Survey study | 2012-2015 | China | 451755 | ≥18 | BP≥140/90 mmHg* | 23.2 | 24.5 | 21.9 | 46.9 | 42.5 | 51.9 | 40.7 | 35.6 | 46.6 | 15.3 | 13.2 | 17.7 |
| Lu, J^10^ | 2017 | China PEACE Million Persons Project | 2014-2015 | China | 1738886 | 35–75 | BP≥140/90 mmHg* | 44.7 | 42.4 | 57.6 | 44.7 | - | - | 30.1 | - | - | 7.2 | - | - |
| Li, Y^11^ | 2017 | 2013-14 CCDRFS survey | 2013-2014 | China | 174621 | >18 | BP≥140/90 mmHg* | 27.8 | 34.5 | 29.5 | 31.9 | 24.2 | 27.5 | 26.4 | 18.5 | 22.0 | 9.7 | 7.2 | 7.9 |
| Wang, C^12^ | 2017 | STEPS | 2011-2013 | Palau | 2529 | 25–64 | BP≥140/90 mmHg* | 46.8 | - | - | - | - | - | - | 14.7 | 31.2 | - | - | - |
| Palafox, B^13^ | 2016 | PURE study | 2006 | China | 46751 | 35–70 | BP≥140/90 mmHg | 41.6 | - | - | 41.7 | - | - | 33.7 | - | - | 8.0 | - | - |
|  |  | PURE study | 2006 | Malaysia | 11825 | 35–70 | BP≥140/90 mmHg | 46.6 | - | - | 48.1 | - | - | 41.2 | - | - | 12.5 | - | - |
|  |  | PURE study | 2006 | Philippines | 1671 | 35–70 | BP≥140/90 mmHg | 51.2 | - | - | 54.5 | - | - | 46.1 | - | - | 13.5 | - | - |
| Peterson, K^14^ | 2016 | 2011–12 AHS | 2011-2012 | Australia | 7269 | ≥18 | BP≥140/90 mmHg | 23.9 | - | - | - | - | - | - | - | - | - | - | - |
| Kessaram, T^15^ | 2015 | STEPS | 2011 | Vanuatu | - | 25–64 | BP≥140/90 mmHg* | - | 32.7 | 30.6 | - | - | - | - | - | - | - | - | - |
|  |  | STEPS | 2010 | French Polynesia | - | 25–64 | BP≥140/90 mmHg* | - | 38.3 | 29.7 | - | - | - | - | - | - | - | - | - |
|  |  | STEPS | 2009 | Wallis and Futuna | - | 25–64 | BP≥140/90 mmHg* | - | 40.7 | 25.8 | - | - | - | - | - | - | - | - | - |
|  |  | STEPS | 2005 | Tokelau | - | 25–64 | BP≥140/90 mmHg* | - | 17.6 | 16.4 | - | - | - | - | - | - | - | - | - |
|  |  | STEPS | 2004 | American Samoa | - | 25–64 | BP≥140/90 mmHg* | - | 43.9 | 32.7 | - | - | - | - | - | - | - | - | - |
|  |  | STEPS | 2004 | Tonga | - | 25–64 | BP≥140/90 mmHg* | - | 26.6 | 21.0 | - | - | - | - | - | - | - | - | - |
|  |  | STEPS | 2002 | Marshall Islands | - | 25–64 | BP≥140/90 mmHg* | - | 19.8 | 19.9 | - | - | - | - | - | - | - | - | - |
|  |  | STEPS | 2005-2006 | Solomon Islands | - | 25–64 | BP≥140/90 mmHg* | - | 13.0 | 13.6 | - | - | - | - | - | - | - | - | - |
|  |  | STEPS | 2004-2006 | Kiribati | - | 25–64 | BP≥140/90 mmHg* | - | 22.8 | 15.1 | - | - | - | - | - | - | - | - | - |
|  |  | STEPS | 2003-2004 | Cook Islands | - | 25–64 | BP≥140/90 mmHg* | - | 43.9 | 32.7 | - | - | - | - | - | - | - | - | - |
|  |  | STEPS | 2011-2012 | Niue | - | 25–64 | BP≥140/90 mmHg* | - | 33.9 | 25.6 | - | - | - | - | - | - | - | - | - |
| Ke, L^16^ | 2015 | - | 2012 | Macao SAR | 1410 | 18–93 | BP≥140/90 mmHg* | 34.0 | 42.0 | 27.0 | 67.0 | 62.0 | 72.0 | 59.0 | 55.0 | 65.0 | 30.0 | 28.0 | 33.0 |
| Wang, J^17^ | 2014 | - | 2009-2010 | China | 50,171 | ≥18 | BP≥140/90 mmHg* | 29.6 | 31.2 | 28.0 | 42.6 | 35.1 | 51.0 | 34.1 | 26.8 | 42.3 | 9.3 | 7.6 | 11.3 |
| Atallah, A^18^ | 2014 | - | - | French Polynesia | 605 | ≥15 | BP≥140/90 mmHg* | 24.5 | - | - | 50.0 | - | - | 32.4 | - | - | 8.8 | - | - |
| Basu, S^19^ | 2013 | SAGE | 2007-2010 | China | 15050 | ≥18 | BP≥140/90 mmHg# | 39.0 | 42.1 | 34.9 | 39.0 | 24.8 | 33.5 | 23.0 | 20.6 | 26.8 | 6.0 | 5.5 | 6.6 |
| Wu, Y^20^ | 2008 | NNHS 2002 | 2002 | China | 218920 | ≥18 | BP≥140/90 mmHg* | 18.0 | 20.0 | 17.0 | - | 25.0 | 22.0 | - | 22.0 | 17.0 | 5.0 | - | - |

*** considered antihypertensive medication use; # considered self-reported diagnosis.**

**Supplementary Figures**

**
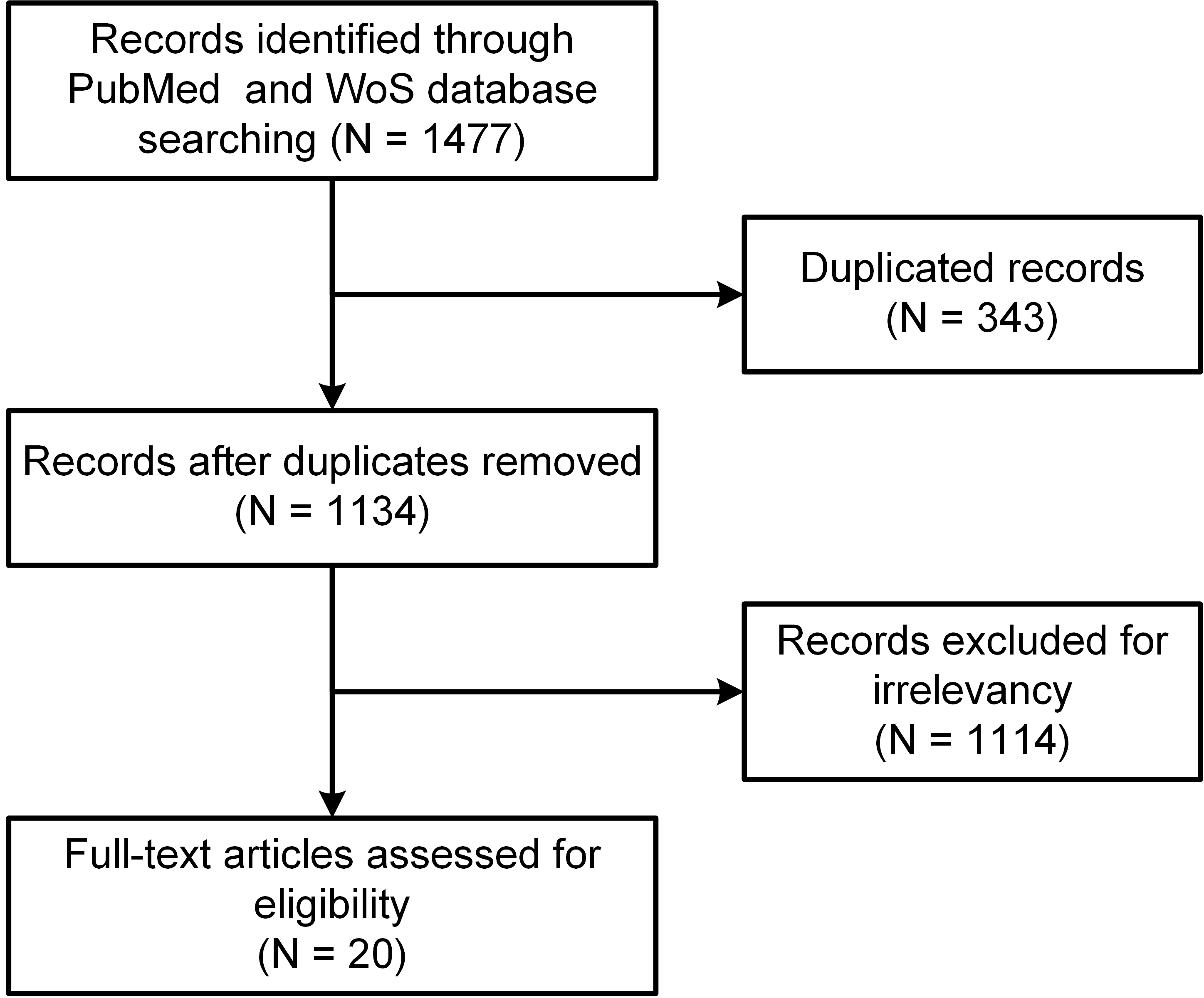
**

**Figure S1. Flowchart of the included** **literature of hypertension prevalence and management.**

**References**

1. Okada A, Yasunaga H. Prevalence of Noncommunicable Diseases in Japan Using a Newly Developed Administrative Claims Database Covering Young, Middle-aged, and Elderly People. *Jma j* 2022; **5**(2): 190-8.

2. Tan MMC, Prina AM, Muniz-Terrera G, et al. Prevalence of and factors associated with multimorbidity among 18 101 adults in the South East Asia Community Observatory Health and Demographic Surveillance System in Malaysia: a population-based, cross-sectional study of the MUTUAL consortium. *BMJ Open* 2022; **12**(12): e068172.

3. An S, Ahn C, Jang J, et al. Comparison of the Prevalence of Cardiometabolic Disorders and Comorbidities in Korea and the United States: Analysis of the National Health and Nutrition Examination Survey. *J Korean Med Sci* 2022; **37**(18): e149.

4. Tafuna'i M, Turner R, Matalavea B, et al. Results of a community-based screening programme for chronic kidney disease and associated risk factors, (obesity, diabetes and hypertension) in a Samoan cohort. *BMJ Open* 2022; **12**(4): e056889.

5. LaMonica LC, McGarvey ST, Rivara AC, et al. Cascades of diabetes and hypertension care in Samoa: Identifying gaps in the diagnosis, treatment, and control continuum - a cross-sectional study. *Lancet Reg Health West Pac* 2022; **18**: 100313.

6. Kirschbaum TK, Theilmann M, Sudharsanan N, et al. Targeting Hypertension Screening in Low- and Middle-Income Countries: A Cross-Sectional Analysis of 1.2 Million Adults in 56 Countries. *J Am Heart Assoc* 2021; **10**(13): e021063.

7. Yu EYT, Yeung CHN, Wan EYF, et al. Association between health behaviours and cardiometabolic dysregulation: a population-based survey among healthy adults in Hong Kong. *BMJ Open* 2021; **11**(7): e043503.

8. Liew SJ, Lee JT, Tan CS, Koh CHG, Van Dam R, Müller-Riemenschneider F. Sociodemographic factors in relation to hypertension prevalence, awareness, treatment and control in a multi-ethnic Asian population: a cross-sectional study. *BMJ Open* 2019; **9**(5): e025869.

9. Wang Z, Chen Z, Zhang L, et al. Status of Hypertension in China: Results From the China Hypertension Survey, 2012-2015. *Circulation* 2018; **137**(22): 2344-56.

10. Lu J, Lu Y, Wang X, et al. Prevalence, awareness, treatment, and control of hypertension in China: data from 1·7 million adults in a population-based screening study (China PEACE Million Persons Project). *Lancet* 2017; **390**(10112): 2549-58.

11. Li Y, Yang L, Wang L, et al. Burden of hypertension in China: A nationally representative survey of 174,621 adults. *Int J Cardiol* 2017; **227**: 516-23.

12. Wang C, Chiang C, Yatsuya H, et al. Descriptive Epidemiology of Hypertension and Its Association With Obesity: Based on the WHO STEPwise Approach to Surveillance in Palau. *Asia Pac J Public Health* 2017; **29**(4): 278-87.

13. Palafox B, McKee M, Balabanova D, et al. Wealth and cardiovascular health: a cross-sectional study of wealth-related inequalities in the awareness, treatment and control of hypertension in high-, middle- and low-income countries. *Int J Equity Health* 2016; **15**(1): 199.

14. Peterson KL, Jacobs JP, Allender S, Alston LV, Nichols M. Characterising the extent of misreporting of high blood pressure, high cholesterol, and diabetes using the Australian Health Survey. *BMC Public Health* 2016; **16**: 695.

15. Kessaram T, McKenzie J, Girin N, et al. Noncommunicable diseases and risk factors in adult populations of several Pacific Islands: results from the WHO STEPwise approach to surveillance. *Aust N Z J Public Health* 2015; **39**(4): 336-43.

16. Ke L, Ho J, Feng J, et al. Prevalence, awareness, treatment and control of hypertension in Macau: results from a cross-sectional epidemiological study in Macau, China. *Am J Hypertens* 2015; **28**(2): 159-65.

17. Wang J, Zhang L, Wang F, Liu L, Wang H. Prevalence, awareness, treatment, and control of hypertension in China: results from a national survey. *Am J Hypertens* 2014; **27**(11): 1355-61.

18. Atallah A, Atallah V, Daigre JL, et al. High blood pressure and obesity: disparities among four French overseas territories. *Ann Cardiol Angeiol (Paris)* 2014; **63**(3): 155-62.

19. Basu S, Millett C. Social epidemiology of hypertension in middle-income countries: determinants of prevalence, diagnosis, treatment, and control in the WHO SAGE study. *Hypertension* 2013; **62**(1): 18-26.

20. Wu Y, Huxley R, Li L, et al. Prevalence, awareness, treatment, and control of hypertension in China: data from the China National Nutrition and Health Survey 2002. *Circulation* 2008; **118**(25): 2679-86.
