# Supplementary material for: Trends and disparities in non-communicable diseases in the Western Pacific region
Source: Lancet Reg Health West Pac. 2023 Dec 1;43:100938. doi: 10.1016/j.lanwpc.2023.100938 (PMC10920054; doi:10.1016/j.lanwpc.2023.100938)
Supplement: Translated Abstract [file mmc2.pdf]

信息页及摘要（中文）

西太平洋地区慢性非传染性疾病的差异和趋势

彭雯 [a,*], 张玲 [b,*], 温馥源 [b], 汤霄 [a],曾令霞 [c], 陈佳鹏 [d], 高力 [e], 闻德亮 [f], 王友发 [c,#]

[a] 青海大学医学部营养与健康促进中心，公共卫生系；青海省中医药防治糖脂代谢病重点实验室
[b] 首都医科大学公共卫生学院；北京市临床流行病学重点实验室
[c] 西安交通大学全球健康研究院，公共卫生学院
[d] 中国人口与发展研究中心
[e] 世界卫生组织驻华代表处
[f] 中国医科大学健康科学研究院

***同等贡献**

# 通讯作者
王友发, 西安交通大学全球健康研究院，公共卫生学院
邮箱: youfawang@gmail.com，youfawang@xjtu.edu.cn 电话: +86-29-82657395

摘要：
西太平洋地区慢性非传染性疾病（简称慢性病）负担重，过去二十年间因慢性病造成的死亡比例呈上升趋势。由于人口快速老龄化、压力增大以及新冠大流行，西太区心理健康疾病负担加重，但这一问题在很大程度上被忽视。高度多样化的文化、宗教、政治制度、社会经济发展、生活方式和环境因素可能导致各国在慢性病死亡率、危险因素和疾病管理方面存在巨大差异和不均衡。慢性病的死亡率存在地理聚集现象，表现为东亚地区的死亡率最低，而太平洋岛国最高。经济繁荣、快速老龄化、营养转型、社会压力、普遍吸烟以及快速增长的肥胖症和高血糖是重要的驱动因素。男性往往具有更多行为和代谢风险因素。农村居民的代谢紊乱增长迅速，正追赶并将超越城市居民。需针对慢性病模式制定可持续的战略，以应对非传染性疾病的流行和相关健康不均衡。

关键词： 慢性非传染性疾病, 趋势, 健康不均衡, 危险因素, 西太平洋地区

Trends and disparities in non-communicable diseases in the Western Pacific region

Wen Peng MD [a,*], Ling Zhang PhD [b,*], Fuyuan Wen [b], Xiao Tang [a], Lingxia Zeng PhD [c], Jiapeng Chen PhD [d], Gauden Galea MD [e], Deliang Wen PhD [f], Youfa Wang PhD [c,#]

[a.] Nutrition and Health Promotion Center, Department of Public Health, Medical College, Qinghai University; Qinghai Provincial Key Laboratory of Prevention and Control of Glucolipid Metabolic Diseases with Traditional Chinese Medicine, No. 16 Kunlun Road, Xining, 810008, People's Republic of China
[b.] School of Public Health, Capital Medical University; Beijing Municipal Key Laboratory of Clinical Epidemiology, No. 10 Xitoutiao, Youanmenwai, Beijing, 100069, People's Republic of China
[c.] Global Health Institute, School of Public Health, Xi'an Jiaotong University, Room 3104, No. 21 Hongren Building, West China Science and Technology Innovation Harbour (iHarbour), Xi'an, 710061, People's Republic of China
[d.] China Population and Development Research Center, No. 12 Dahuisi Road, Beijing, 100081, People's Republic of China
[e.] WHO China Representative Office, No.23 Dongzhimenwai Street, Beijing, 100600, People's Republic of China
[f.] Health Sciences Institute, China Medical University, No.77 Puhe Road, Shenyang North New Area, Shenyang, Liaoning Province, People's Republic of China

Brief title: Trends and disparities in NCDs

***Equal contribution**

# Corresponding author
Youfa Wang, MD, PhD, MS
Global Health Institute
Xi'an Jiaotong University Health Science Center
Room 3104, No. 21 Hongren Building
West China Science and Technology Innovation Harbour (iHarbour)
Xi'an, Shaanxi, China.
Email: youfawang@gmail.com, youfawang@xjtu.edu.cn
Telephone: +86-29-82657395

Summary:
The WHO Western Pacific region bears disproportionate deaths from non-communicable diseases (NCDs), with increased overall NCD proportional mortality over the past two decades. Disease burden of mental health increased, resulting from rapid aging, enhanced stress, and COVID-19 pandemic, but it was largely neglected. The highly diverse cultures, religions, political systems, socioeconomic contexts, lifestyles, and environmental factors probably have led to massive disparities across countries in NCD mortality, risk factors, and NCD management. Geographically, East Asia had the lowest NCD mortality whilst Pacific islands the highest. Economic booms, rapid ageing, nutrition transition, social stress, prevalent tobacco use, and fast increasing obesity and hyperglycemia are important drivers of NCDs. Men tended to have more adverse behavioral and metabolic risk factors. Rural residents are catching up with their urban counterparts in metabolic risk factors and conditions. Sustainable strategies tailored at NCD patterns are needed to fight the NCD epidemic and related disparities.

Keywords: non-communicable diseases, trends, disparity, risk factors, the Western Pacific region
